# Prospective insight into the role of benzyl propylene glycoside as a modulator of the cGAS-STING signaling pathway in the management of nonalcoholic fatty pancreas animal model

**DOI:** 10.1186/s40659-023-00423-8

**Published:** 2023-03-13

**Authors:** Reda Albadawy, Amany Helmy Hasanin, Sara H. A. Agwa, Shaimaa Hamady, Reham Hussein Mohamed, Eman Gomaa, Mohamed Othman, Yahia A. Yahia, Amani Mohamed Abdel Ghani, Marwa Matboli

**Affiliations:** 1 Department of Gastroenterology, Hepatology & Infectious Disease, Faculty of Medicine, Benha University, Benha, 13518 Egypt; 2 Clinical Pharmacology Department, Faculty of Medicine, Ain Shams University, Cairo, Egypt; 3 Clinical Pathology and Molecular Genomics Unit, Medical Ain Shams Research Institute (MASRI), Faculty of Medicine, Ain Shams University, Cairo, 11382 Egypt; 4 Department of Biochemistry, Faculty of Science, Ain Shams University, Cairo, 11566 Egypt; 5 Histology and Cell Biology Department, Faculty of Medicine, Ain Shams University, Giza, Egypt; 6 Gastroenterology and Hepatology Section, Baylor College of Medicine, Houston, TX 77030 USA; 7 Chemistry Department, School of Science and Engineering, American University in Cairo, New Cairo, 11835 Egypt; 8 Biochemistry Department, Faculty of Pharmaceutical Sciences and Drug Manufacturing, Misr University for Science and Technology, Giza, Egypt; 9 Clinical Pathology, Faculty of Medicine, Ain Shams University, Cairo, 11566 Egypt; 10 Medical Biochemistry and Molecular Biology Department, Faculty of Medicine, Ain Shams University, Cairo, 11566 Egypt

**Keywords:** Nonalcoholic fatty pancreas, cGAS-STING pathway, Benzyl propylene glycoside, Rats, Obesity

## Abstract

**Background:**

Nonalcoholic fatty pancreatitis (NAFP) is one of the metabolic syndrome manifestations that need further studies to determine its molecular determinants and find effective medications. We aimed to investigate the potential effect of benzyl propylene glycoside on NAFP management via targeting the pancreatic cGAS-STING pathway-related genes (DDX58, NFκB1 & CHUK) and their upstream regulator miRNA (miR-1976) that were retrieved from bioinformatics analysis.

**Methods:**

The rats were fed either normal chow or a high-fat high-sucrose diet (HFHS), as a nutritional model for NAFP. After 8 weeks, the HFHS-fed rats were subdivided randomly into 4 groups; untreated HFHS group (NAFP model group) and three treated groups which received 3 doses of benzyl propylene glycoside (10, 20, and 30 mg/kg) daily for 4 weeks, parallel with HFHS feeding.

**Results:**

The molecular analysis revealed that benzyl propylene glycoside could modulate the expression of the pancreatic cGAS-STING pathway-related through the downregulation of the expression of DDX58, NFκB1, and CHUK mRNAs and upregulation of miR-1976 expression. Moreover, the applied treatment reversed insulin resistance, inflammation, and fibrosis observed in the untreated NAFP group, as evidenced by improved lipid panel, decreased body weight and the serum level of lipase and amylase, reduced protein levels of NFκB1 and caspase-3 with a significant reduction in area % of collagen fibers in the pancreatic sections of treated animals.

**Conclusion:**

benzyl propylene glycoside showed a potential ability to attenuate NAFP development, inhibit pancreatic inflammation and fibrosis and reduce the pathological and metabolic disturbances monitored in the applied NAFP animal model. The detected effect was correlated with modulation of the expression of pancreatic (DDX58, NFκB1, and CHUK mRNAs and miR-1976) panel.

**Supplementary Information:**

The online version contains supplementary material available at 10.1186/s40659-023-00423-8.

## Background

Although non-alcoholic fatty pancreas (NAFP) was reported early in the 1930s, our knowledge about this disease is still in its infancy and perceived as a relatively new condition [1]. NAFP is described as pancreatic fat cumulation without significant alcohol intake [2]. It was considered a benign incidental finding, and therefore its clinical consequences were ignored. The prevalence of NAFP ranges from 16 to 35% and is increasingly associated with obesity, insulin resistance (IR), deterioration of beta-cell function and metabolic syndrome which might lead to the development of diabetes and pancreatitis [1]. Therefore, its early detection may help to diagnose prediabetic patients to reduce the rising morbidity and mortality due to diabetes mellitus.

Although the shared association between NAFP and non-alcoholic fatty liver disease (NAFLD), the implicating mechanisms still unclear and has led researchers to hypothesize comparable etiologies of NAFP and NAFLD [3]. The metabolic stress in NAFP, including insulin resistance and obesity, can stimulate severe acinar cell injury resulting in progressive acinar cell death, and acute pancreatitis, which acts as a trigger for various signaling mechanisms including the cyclic GMP-AMP synthase (cGAS)-stimulator of interferon genes (STING) pathway [4]. The cGAS-STING pathway was found to be activated in acute pancreatitis and can induce cell injury by activating inflammation and by disturbing glucose and lipid metabolism [5, 6]. STING activation also affects several signaling cascades resulting in the induction of the nuclear factor kappa beta (NF-kB) to produce proinflammatory cytokines and activate fibrogenesis [7]. Therefore, more exploration into this signaling mechanism might help in identifying novel therapies for NAFP disease.

Dysregulation of microRNA (miRNA) may impact the function and status of various tissues, like the pancreas [8–10], and liver [11–13], contributing to metabolic disorders associated with obesity and insulin resistance-linked diseases including NAFP. miRNAs play a very important role as key regulators of inflammation, insulin signaling, and glucose and lipid metabolism. However, information about the mechanisms of their implication in NAFP progression remains nearly limited, due to the ability of miRNAs to simultaneously affect several gene/pathway networks [14]. This integrated gene (mRNAs)—miRNAs regulatory interaction may provide new early non-invasive diagnostic biomarkers and identification of therapeutic strategies for NAFP. Obviously, bioinformatic analysis facilitates the identification of new candidate RNA species and their interactions as biomarkers for disease screening, diagnosis, and therapy [15].

Emerging studies have reported that benzyl propylene glycoside (Rosavin), a main constituent of the Rhodiola Rosea plant, possesses several pharmacological effects such as anti-inflammatory, anti-adipogenic and hepato-protective effects on metabolic syndrome and related disorders [16–18]. The underlying mechanisms behind these effects may involve inhibition of NF-kB, reducing cell death, inhibition of adipogenesis, and modulation of miRNA expression [19], and this suggests that the miRNA may be a target for benzyl propylene glycoside treatment. However, its effect on NAFP is not clearly illustrated.

Based on the all previously discussed data, we aimed to investigate the potential therapeutic efficacy of benzyl propylene glycoside on NAFP management via targeting the pancreatic cGAS-STING pathway-related genes (DDX58, NFκB1 & CHUK) and their upstream regulator miRNA (miR-1976) that were retrieved from bioinformatics analysis in NAFP animal model.

## Results

### Benzyl propylene glycoside—miR-1976 in-silico interaction prediction

**Fig. 1:**
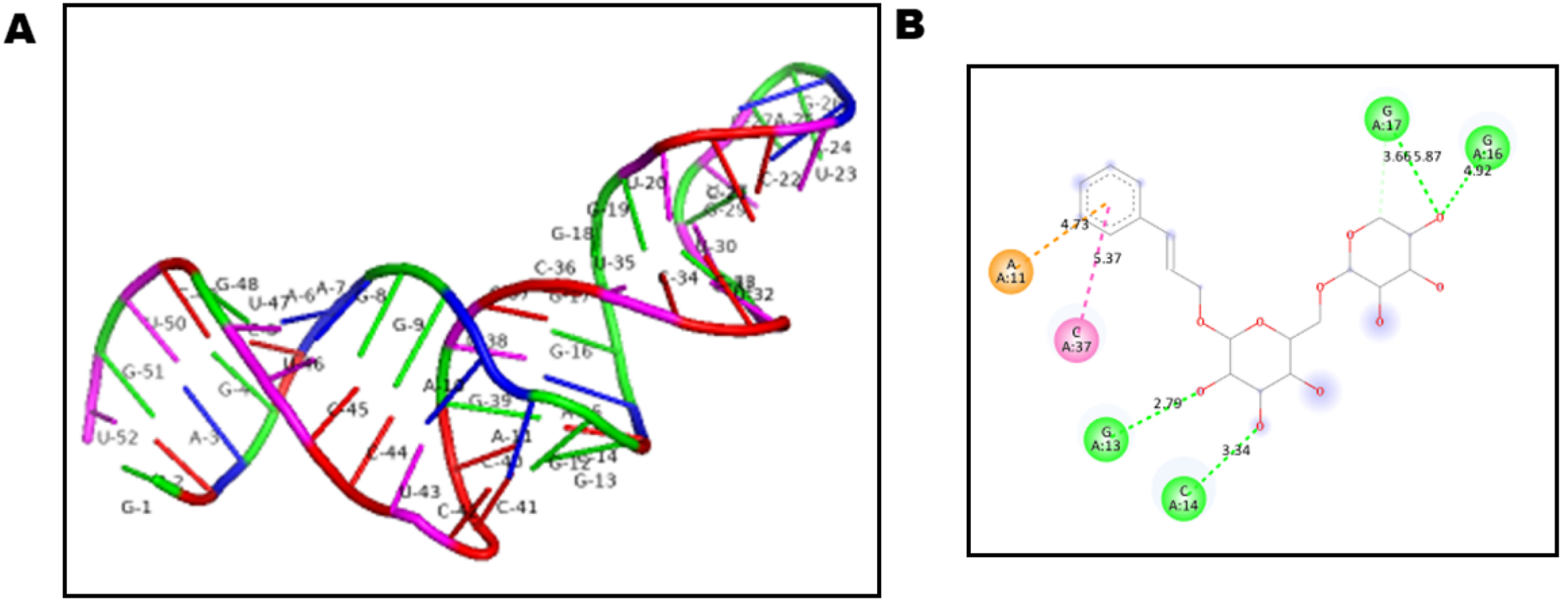
**A** 3D structure of miR-1976 as predicted. U: Uridine, G: Guanosine, C: Cytosine, and A: Adenosine. **B** Benzyl propylene glycoside-miR-1976 ranked 1 interaction. Green interaction: Hydrogen bonding. Pink interaction: T-shaped pi–pi interaction. Orange interaction: pi-pi anionic interaction

The miRNA1976 secondary structure modelling showed MFE for thermodynamic ensemble of -31.85 kcal/mol which was further used to obtain the 3D model (Fig. 1A). Docking scores were recorded as in Table 1. Benzyl propylene glycoside (Rosavin) predicted interactions were sketched as an interaction per nucleotide and type of bond for the top 10 poses (Additional file 1: Fig. S1). HDock calculated confidence score was over 0.5 for one pose with a docking score of − 151.9 and a calculated RMSD of 40.24 (Fig. 1B).

**Table 1:** Rosavin-miRNA1976 docking poses as computed by HDock server

Rank	1	2	3	4	5	6	7	8	9	10
Docking score	− 151.90	− 148.36	− 148.21	− 147.51	− 146.05	− 145.32	− 142.85	− 141.85	− 141.14	− 139.81
Confidence score	0.5095	0.4918	0.4911	0.4876	0.4803	0.4766	0.4643	0.4593	0.4558	0.4492
Ligand RMSD (Å)	40.24	15.72	13.81	43.41	12.38	11.79	38.33	10.39	41.62	9.85

### Effect of 8 weeks of HFHS feeding on body weight and blood biochemical parameters

Feeding experimental rats with an HFHS diet for 8 weeks (Table 2) has resulted in a significant increase (p < 0.001) in body weight, HbA1C%, the levels of lipid profile markers, fasting serum glucose, and insulin as well as insulin resistance represented by HOMA-IR, compared to the Sham animals. In addition, a significant elevation in the level of serum amylase and lipase was also recorded in HFHS-fed animals. The results indicated that the HFHS-challenged animals exhibited signs of dyslipidemia and pancreatic damage.

**Table 2:** Body weight and blood parameters after 8 weeks of HFHS feeding

	Sham	HFHS-8 week
Initial body weight, g	160.3 ± 8.08	164.7 ± 8.96
Final body weight, g	190.8 ± 8.08	355.3 ± 9.33^*^
TC (mg%)	123.8 ± 11.68	227.3 ± 7.076^*^
TG (mg%)	49.07 ± 4.53	145.5 ± 13.6^*^
HDL (mg%)	59.33 ± 3.64	38.07 ± 2.84^*^
LDL (mg%)	54.27 ± 12.74	160.3 ± 7.86^*^
Glucose (mg%)	100.7 ± 11.07	280.3 ± 28.08^*^
Insulin (μU/ml)	4.907 ± 0.89	15.08 ± 1.16^*^
HOMA-IR	1.223 ± 0.26	10.42 ± 1.160^*^
HbA1C%	4.28 ± 0.80	9.94 ± 0.91^*^
Lipase (U/L)	354 ± 52.89	3523 ± 441.8^*^
Amylase (U/L)	1006 ± 94.21	9143 ± 1221^*^

Values are mean ± SD; number = 15 rats/each group. Obtained from sample t-test

^*^p < *0.001* vs Sham

### Effect of benzyl propylene glycoside treatment on body weight and blood biochemical parameters

As shown in Table 3, body weight was significantly higher at the end of 12 weeks in untreated HFHS-fed rats than in Sham animals. After 4 weeks from benzyl propylene glycoside treatment, the body weight was significantly attenuated in HFHS rats (R-20 & R-30) compared to the NAFP group. Feeding animals an HFHS diet for the entire 12 weeks (NAFP group) caused a significant upsurge in the levels of serum glucose, insulin, and HbA1C% compared to the normal chow-fed rats (Sham group). Therefore, untreated NAFP rats presented a higher HOMA-IR (p < 0.001) than those of the Sham group. Serum levels of TG, TC, and LDL-C, but not HDL-C, also significantly (p < 0.001) increased in NAFP animals compared to the animals of the Sham group. On the other hand, animals of the three treated groups displayed a significant correction in all previous variables compared to the untreated NAFP group in a dose-dependent manner.

**Table 3:** Ingredients, and energy content of the normal chow and high-fat and high-sucrose (HFHS) diets [22]

Diet ingredients, g/kg	Normal chow	HFHS
Lard	–	180
Sucrose	100	300
Casein	140	160
Starch	620.7	220.7
Fiber	50	50
Cholic acid	2.5	2.5
Soybean oil	40	40
Vitamin mix & Mineral mix	45	45
L-cysteine	1.8	1.8
Energy kcal/g	3.81	4.71
Carbohydrate %	75.7	44.2
Protein %	14.9	13.7
Lipid %	9.4	42.0

Regarding serum amylase and lipase, a highly significant elevation in the levels of these enzymes was recorded in animals in the untreated NAFP group compared to the Sham group. Daily injection with benzyl propylene glycoside for four weeks, caused a significant reduction in the levels of serum amylase and lipase, in comparison with the NAFP group. This ameliorative effect was more prominent in both the R-20 and R-30 groups.

As compared to the HFHS-8 week group, the benzyl propylene glycoside-treated groups (R-20 and R-30) showed a significant reduction in all previously mentioned biochemical variables that indicated Benzyl Propylene Glycoside has the potential to restore the initial pathological changes induced by HFHS feeding. Moreover, the results demonstrated that the four additional weeks of the HFHS diet resulted in more severe damage in NAFP animals.

### Histological observations

**Fig. 2:**
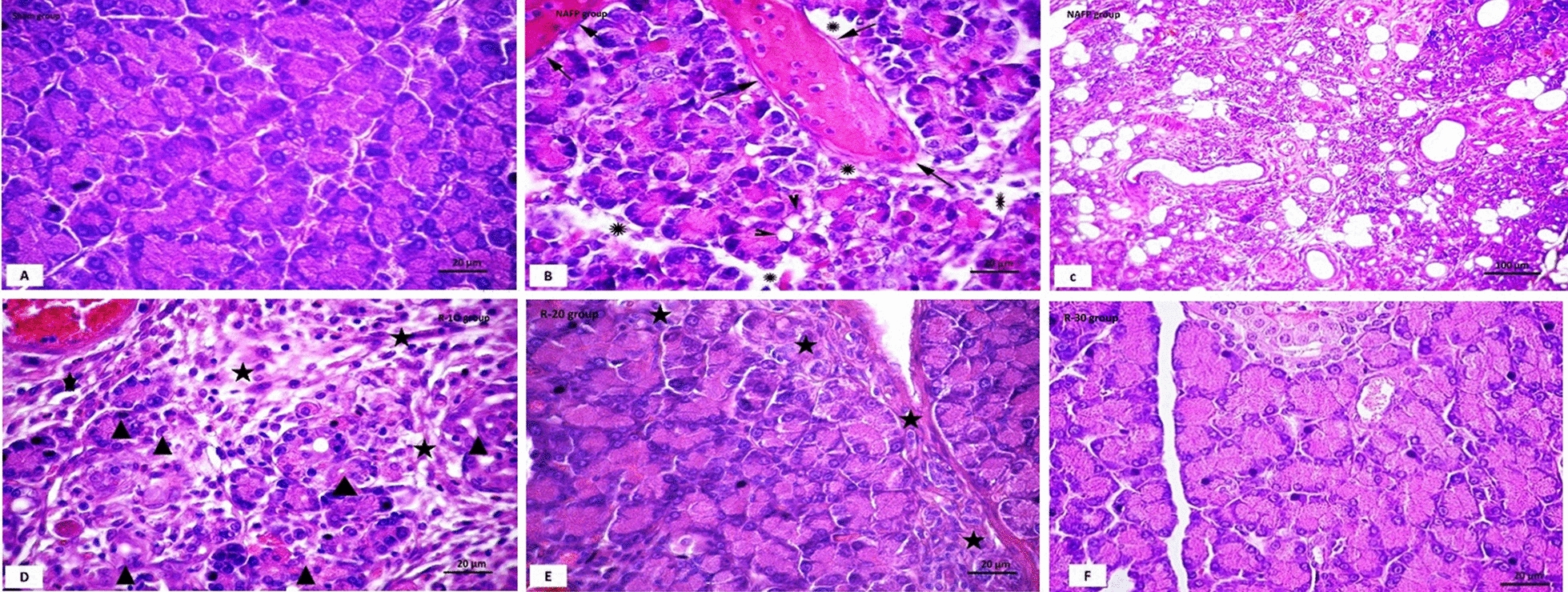
H&E-stained pancreas sections of **A**; Sham group showed closely packed pancreatic acini with basal basophilia and apical acidophilia and the acinar cells have basal open phase nuclei, **B**, **C**; NAFP group showed loss of pancreatic architecture, fat deposition among distorted acini (f), a congested and dilated blood vessel (↑), and fibrin clot with inflammatory cells margination and pavementation. The adjacent pancreatic acini were distorted, and some acinar cells showed vacuolated cytoplasm and pyknotic nuclei (▲) with edematous clear areas in-between the acini (*), **D**; R-10 group showed focal areas of loss of architecture and the acini in the affected areas showed variable structural changes. Some acini attained lightly stained cells with loss of basal basophilia and apical acidophilia and other acinar cells showed vacuolated cytoplasm and pyknotic nuclei (▲) with area of oedema and inflammatory cell infiltration (*), **E**; R-20 group showed the pancreatic lobules and the pancreatic acini are closely packed. Thin interlobular septa can be seen. There were focal areas of structural changes at the periphery of pancreatic lobules, where some acini attained pale vacuolated cytoplasm, (*), and **F**; R-30 group showed most of the pancreatic acini attained numerous and closely packed zymogen granules and the nuclei are basal and vesicular. [Magnification: 200x]

The light microscopic examination of H&E sections of the pancreas of the Sham group revealed normal acinar arrangement with basal basophilia and apical acidophlia and the acinar cells have basal open phase nuclei (Fig. 2). The NAFP group showed loss of the normal lobulation of the pancreas. Large areas of pancreatic parenchyma were occupied by fat cells with noticeable areas of fat necrosis. Some intact acini were seen in between fat tissue. Meanwhile, the remaining acini appeared distorted, vacuolated. Rounded structures of variable size were detected which might be regenerating acini. Areas of intense mononuclear cellular infiltration and oedema were also noticed. The interlobular and intralobular connective tissues were relatively thickened. Some blood vessels showed fibrin clots with margination and pavementation of inflammatory cells. While pancreas of R-10 group showed focal structural changes in some lobules as well as interlobular and intralobular connective tissue. The affected acinar cells showed variable structural changes. Some acinar cells were lightly stained with loss of basal basophilia and apical acidophilia. Others showed vacuolated cytoplasm with pyknotic nuclei within oedematous areas. In group R-20, the pancreatic lobules and the pancreatic acini were closely packed, however, noticeable areas of pancreatic affection were still present. There were focal areas of disorganized acini. Localized areas of mononuclear cellular infiltration were still noticed. In R-30 group, pancreas showed normal structure with tightly packed pancreatic acini and thin interlobular septa. Most acini were formed of normal acinar cells with basal basophilia and apical secretory granules and vesicular nuclei. Few acini showed hyalinized cytoplasm.

**Fig. 3:**
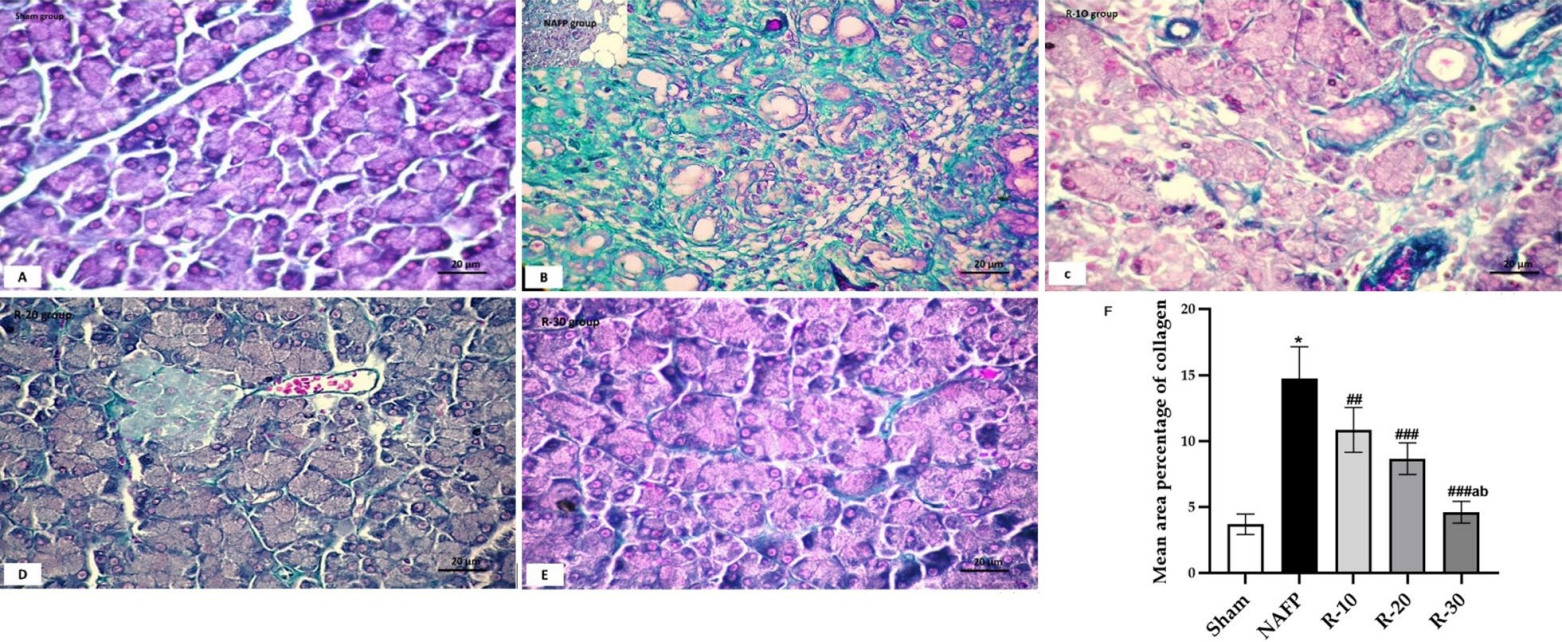
Masson trichrome-stained pancreas sections of **A**; Sham group showed minimal green color of collagen fibers in between the closely packed pancreatic acini, **B**; NAFP group showed marked green collagen fibers deposition in the interlobular septa and in between the destructed acini, **C**; R-10 group collagen fibers deposition in the interlobular septa and in between the destructed acini, **D**; R-20 group showed collagen fibers especially around blood vessels and distorted acini, and **E**; R-30 group showed mild collagen fibers deposition around acini. [Magnification: 200x]. **F** The mean area % of collagen deposition (± SD) in the Sham and the experimental groups (n = 6): ^*^P < *0.001* vs the Sham group; ^*###*^*P* < 0.001, and ^*##*^*P* < 0.01 vs NAFP group. ^*a*^*P* < 0.05 vs R-10. ^*b*^*P* < 0.05 vs R-20. Measurements were taken from three different sections obtained from each animal. Moreover, five haphazardly selected non-overlapping fields were examined for each section

In Masson’s trichrome stained sections (Fig. 3) showed progressive increase of collagen fibers deposition in all groups to be maximum in NAFP. In the treated group, collagen fibers were still noticed surrounding the blood vessels and thickened interlobular septa in both groups R-10 and R-20, but they were more pronounced in R-10. Despite of that, collagen fibers were apparently less than those in R-30. These results were confirmed by the statistical study. Morphometric and statistical study for area percentage of collagen fibers (Fig. 3F) revealed significant increase in NAFP in relation to other groups. In the treated group R-10 there was a significant increase as compared to the control. However, R-20 showed significant decrease as compared to NAFP group.

### The effect of benzyl propylene glycoside on the expression of the pancreatic selected RNA species

**Fig. 4:**
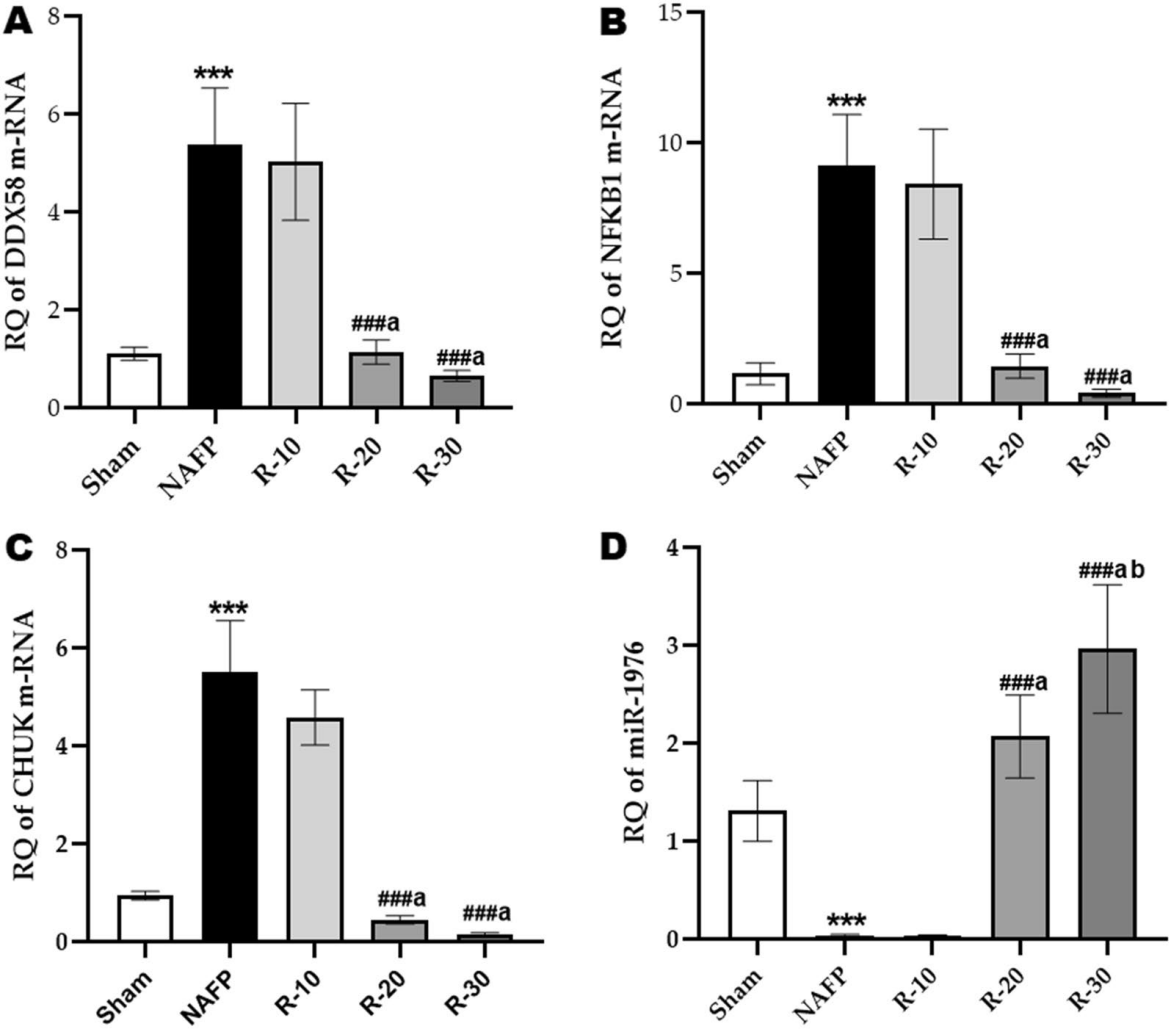
Effect of benzyl propylene glycoside on the expression of the pancreatic selected RNA species **A** DDX58. **B** NFκB1 **C** CHUK. **D** miR-1976. Values are mean ± SD; n = 8 rats/each group. ^***^P < 0.001 and ^**^P < 0.01 vs Sham group; ^*###*^*P* < 0.001 vs NAFP group. ^*a*^*P* < 0.05 vs R-10. ^*b*^*P* < 0.05 vs R-20. One-way ANOVA followed by Tukey’s multiple comparison test RQ, relative quantification

Results showed a significant elevation in the expression of pancreatic DDX58, NFκB1 and CHUK mRNAs with a significant reduction in the expression level of miR-1976 in the untreated NAFP group compared to the Sham group *(p* < *0.001)*, Fig. 4. Meanwhile, the administration of benzyl propylene glycoside at its two higher dosages 20 & 30 (R-20 and R-30) significantly reduced the significant upregulation in the expression of pancreatic mRNA species manifested in untreated NAFP group animals. Moreover, the data were coupled with a significant increase in the expression of miR-1976 in the treated groups (R-20 and R-30) compared to the untreated NAFP group.

### The effect of benzyl propylene glycoside on the pancreatic NFκB1 and Caspase-3

**Fig. 5:**
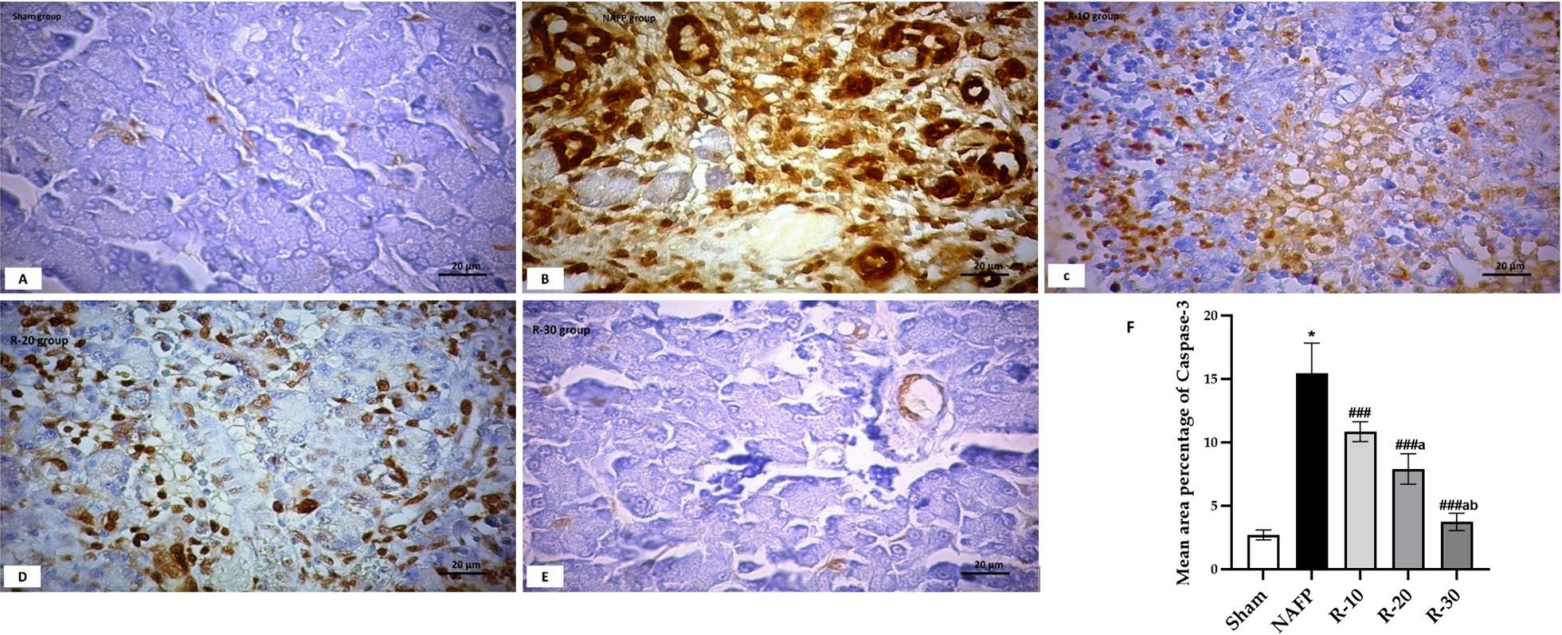
**A**–**E** Caspase-3 immunohistochemistry-stained pancreas sections of **A**; Sham group showed minimal reaction for caspase-3 among acinar cells, **B**; NAFP group showed extensive positive reaction for caspase-3, **C**; R-10 group showed positive reaction for caspase-3 in destructed areas, **D**; R-20 group showed moderate positive reaction for caspase-3 in destructed areas, and **E**; R-30 group showed minimal positive reaction for caspase-3. [Magnification: 200x]. **F**; The mean area percentage of Caspase-3 positive cells (± SD) in the Sham and the experimental groups (n = 6): ^*^P < *0.001 vs* the Sham group; ^*###*^*P* < 0.001 vs NAFP group. ^*a*^*P* < 0.05 vs R-10. ^*b*^*P* < 0.05 vs R-20

As shown in Fig. 5A–E, caspase-3-stained sections revealed minimal reaction in acinar cells in R-30 group. However moderate reaction was noticed in both the cytoplasm of acinar cells and the rounded structures in R-20. Positive reaction was distinguished in most of acinar cells in NAFP group. Morphometric and statistical study for area percentage of Caspase-3 positive cells reaction revealed significant elevation in NAFP and R-10 in comparison to other groups.

**Fig. 6:**
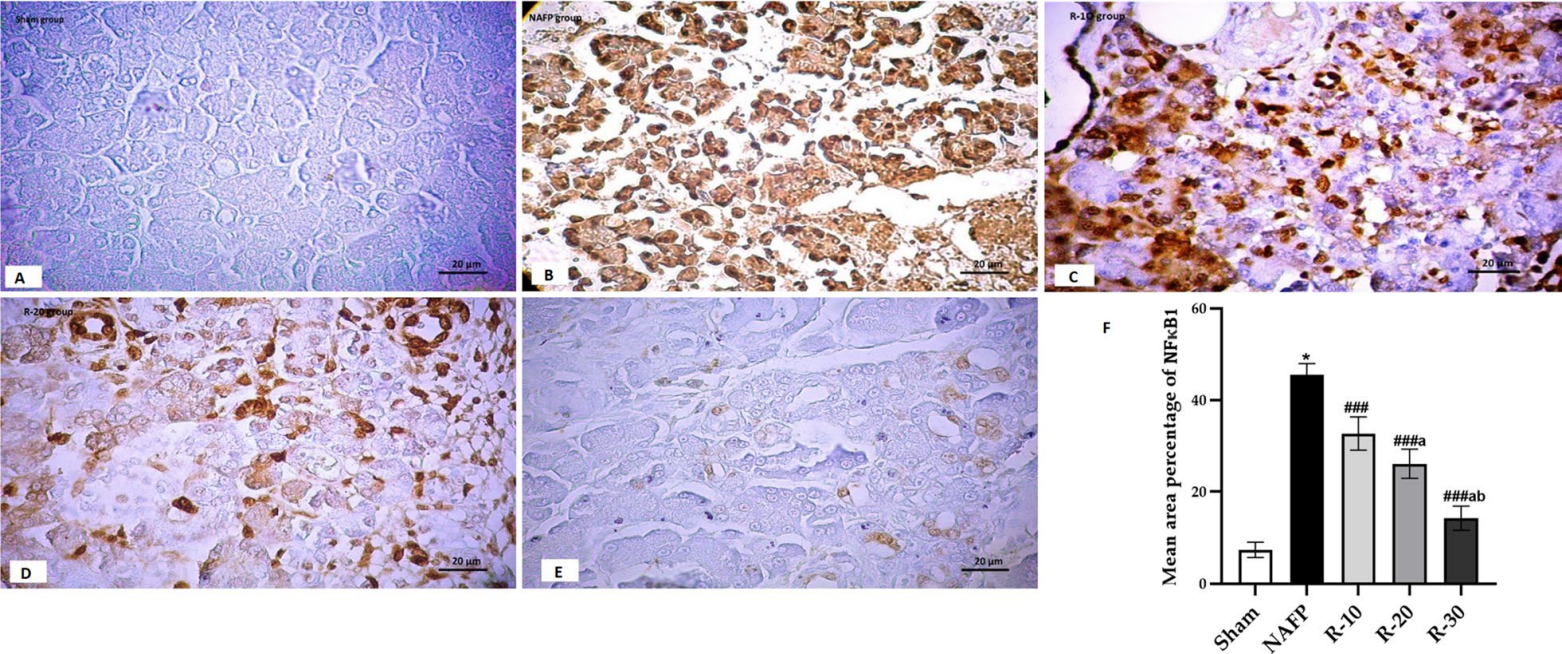
**A**–**E** NFκB1 immunohistochemistry-stained pancreas sections of **A**; Sham group showed minimal reaction for NFκB1 among acinar cells, **B**; NAFP group showed extensive positive reaction for NFκB1, **C**; R-10 group showed positive obvious reaction for NFκB1 among distorted pancreatic acini, **D**; R-20 group showed moderate positive reaction for NFκB1 in destructed areas, and **E**; R-30 group showed minimal positive reaction for NFκB1. [Magnification: 200x]. **F**; The mean area percentage of NFκB1 positive cells (± SD) in the Sham and the experimental groups (n = 6): ^*^P < *0.001 vs* the Sham group; ^*###*^*P* < 0.001 vs NAFP group. ^*a*^*P* < 0.05 vs R-10. ^*b*^*P* < 0.05 vs R-20

Nuclear factor kappa-stained sections (Fig. 6A–E) revealed minimal reaction in acinar cells in R-30 group. However moderate reaction was noticed in both the acinar cells and the rounded structures in R-20. Maximum Positive reaction was distinguished in most cells in NAFP group. Morphometric and statistical study for area percentage of nuclear factor kappa stained \ cells revealed significant elevation in R-10 group in comparison to other treated groups.

## Discussion

Nonalcoholic fatty pancreatitis (NAFP) considers one of the manifestations of metabolic syndrome that needs further studies to determine molecular determinants of this disorder and find effective medications [1]. Emerging data showed that insulin resistance and dysregulation of the cyclic GMP-AMP synthase (cGAS)-stimulator of interferon genes (STING) pathway are the major driving forces for acute pancreatitis and fibrogenesis in NAFP progression [5]. Thus, herein, we constructed a mRNAs (DDX58, NFκB1& CHUK)—(miR-1976) panel linked to metabolic syndrome and pancreatic cell dysfunction as well as be enrolled in the cGAS-STING pathway via in silico data analysis. Then we evaluated the potential ameliorative effects of benzyl propylene glycoside (Rosavin) treatment, the main constituent of the Rhodiola Rosea plant, on NAFP management and its effects on the constructed RNA panel in the NAFP animal model.

One of the primary mechanisms that explain the incidence of the fatty pancreas is the infiltration of adipocytes into the pancreatic tissue. Obesity and increased body weight are the major contributing factors to this condition. Adipose tissue is an endocrine organ as it emits signals to different organs. During weight gain, the storage of fat in adipose tissue is overridden, resulting in the excess lipid is deposited in visceral and peripheral non-adipose organs including the pancreas [20]. Therefore, fatty infiltration of the pancreas is detected as ectopic adipocytes infiltrating the pancreatic tissue where fats deposit in adipocytes in the pancreatic tissue inducing pathological disorders such as insulin resistance and pancreatic cell injury and ultimately resulting in pancreatitis [21].

Accordingly, all previously discussed data can illustrate the results we obtained. We have used a high-fat and high-sucrose (HFHS) feeding as a representative experimental animal model of NAFP disease. Accumulating studies have investigated the impacts of HFHS diet on experimental animals, and it has been concluded that consumption of this diet induces obesity and insulin resistance [22–24]. In the current study, this nutritional model nearly covered the spectrum of the pathological and metabolic disturbances associated with NAFP. The HFHS diet feeding resulted in increased body weight, hyperglycemia, hyperinsulinemia, insulin resistance, and dyslipidemia in the untreated NAFP group. The animals also showed degrees of pancreatitis manifested by large areas of fat cells with noticeable areas of fat necrosis as well as areas of intense mononuclear cellular infiltration and oedema were also noticed in the pancreatic sections, and elevated serum levels of amylase and lipase.

Recently, it was reported that the cyclic GMP-AMP synthase (cGAS)-stimulator of interferon genes (STING) pathway can be activated by lipotoxicity-induced pancreatic cell injury [25]. cGAS-STING signaling pathway is a crucial regulator of immune responses [26] and plays an important role in glucolipid metabolic disorders and was found to be activated in the animals fed a high-fat diet, and its gene silencing reversed metabolic dysfunction, insulin resistance, and inflammation [27]. Activated STING can stimulate the phosphorylation of interferons (IRFs). Phosphorylated IRF regulates the expression of target genes, including DEAD Box Protein 58 (DDX58) & Nuclear Factor Kappa B Subunit 1 (NFκB1), to activate diverse downstream signaling pathways and promote the expression of inflammatory and fibrotic genes [25]. Therefore, STING can promote cellular inflammation in several pathological conditions like insulin resistance.

The current results are consistent with the previously discussed data where a significant increase in the expression level of the pancreatic cGAS-STING pathway-related genes (*DDX58* and *NF*κ*B1*) in the untreated HFHS-fed animals (NAFP group) compared to the Sham group (p < 0.001). The results were propped by the results of histological and immunohistochemistry assay which showed a significant prevalent maximum positive immunostaining for NFκB1 marker, coupled with large areas of inflammatory cells infiltration in the pancreatic tissue, in comparison with the Sham group. Parallel studies have also confirmed that *NF*κ*B1* and *DDX58* are upregulated in pancreatic cell injury [28–30]. Moreover, DDX58 was reported to be one of the genes that were differentially expressed in obese patients with type two diabetes mellitus [31].

The NF-KB activation requires the activity of the upstream serine/threonine protein kinase alpha (IKKα) which is encoded by the CHUK (conserved helix-loop-helix ubiquitous kinase) gene. The phosphorylation of nuclear factor-kB inhibitor (IKB) by IKKα results in its degradation and activation of NFκB1 [32]. The results of the present study revealed a significant upregulation in the expression of the pancreatic *CHUK* in the NAFP group compared to the Sham group.

Increased expression of caspase-3 has been observed in the absence of apoptosis. Caspase-3 may be implicated in processes other than apoptosis where it can participate in inflammatory responses by cleaving and activating cytokines [33]. Moreover, it was also reported that increased caspase-3 in the high-fat diet-fed animal was associated with a significant elevation in hepatic expression of inflammatory cytokines indicating that increased apoptosis could be an insulting mechanism in hepatic inflammation [34]. Consistently, our results displayed a marked increase in caspase-3 protein expression in the pancreas of NAFP model rats compared to the Sham group.

Herein, all observed disturbances in the untreated NAFP animals were significantly adjusted by treatment of the experimental animals with benzyl propylene glycoside daily for four weeks. The recent studies on benzyl propylene glycoside showed that it exhibits anti-oxidative [35], anti-cancer [36], and anti-inflammatory effects [37]. The toxicity of benzyl propylene glycoside has been previously assessed and shown to have a hepatoprotective effect and can alleviate kidney damage [17, 38, 39]. An emerging study that evaluated the toxicity of Rhodiola components showed that LD50 > 5000 mg/kg b.w., considers safe for consumption. This study also showed that long-term administration of Rhodiola doses (100, 250, and 500 mg/kg b.w.) for 28 days didn’t cause any toxic effects in experimental animals. Moreover, all the parameters related to the liver, and kidney were not affected [40].

It was also reported that benzyl propylene glycoside can attenuate cell injury and fibrosis through inhibition of NF-kB and decreasing the production of pro-inflammatory and fibrotic cytokines [41]. Benzyl propylene glycoside can also improve cellular immunity by inhibiting tissue apoptosis [37].

The results of the current study were in accordance with the published data. The results revealed that the daily treatment with benzyl propylene glycoside had beneficial actions on the progression of NAFP. It significantly improved the lipid panel, decreased the body weight, lowered the serum insulin and glucose levels, ameliorated the insulin resistance status, and decreased the serum level of lipase and amylase. Surprisingly, as compared to the HFHS-8 week group, the benzyl propylene glycoside-treated groups (R-20 and R-30) revealed significant decreases in all detected biochemical variables that showed benzyl propylene glycoside has the potential to prevent the progression of exocrine pancreatic damage and can recover the initial pathological changes induced by HFHS feeding. Moreover, the applied treatment decreased the expression level of cGAS-STING pathway-related genes, DDX58, NFκB1 & CHUK, coupled with decreased the protein expression of pancreatic inflammatory NFκB1 and caspase-3 as compared with the untreated NAFP animals. Normal structure with tightly packed pancreatic acini and thin interlobular septa with a significant decrease in area percentage of collagen fibers were also detected in the treated groups (R-30) when compared to the NAFP model group. The revealed results indicate that benzyl propylene glycoside could improve pancreatic tissue injury via modulating and inhibiting the cGAS-STING pathway.

The currently identified biomarkers in the early diagnosis of NAFP are insufficient and poorly known. Thus, novel non-invasive biomarkers and precise therapeutic targets are required urgently. MicroRNAs (miRNAs) are a class of small non-coding RNAs that modulate the expression of protein-coding genes [42]. They can be detected in body fluids, like blood and urine, and changes in their levels have been associated with several diseases therefore they can be utilized as diagnostic biomarkers [43]. Accumulating evidence shows that modulation of miRNA expression could be one of the regulatory mechanisms behind the ameliorative activities of benzyl propylene glycoside [19]. miRNAs also play crucial roles in the function and survival of pancreatic cells and have been found to regulate the adaptive responses of pancreatic cells in conditions like obesity and pancreatitis [42, 44].

In the present study, the miRWalk database was utilized to retrieve the upstream regulators, miR-1976, for the selected three mRNAs (CHUK, NFκB1, and DDX58). Regarding the in-silico study of benzyl propylene glycoside-miR-1976 interaction, the confidence scores showed slight significance for successful binding for the first pose with a confidence score > 0.5. However, the high deviation in the RMSD suggests the decreased probability of binding which needs further experimental proof of direct binding [45]. Nevertheless, the alteration of miR-1976 maybe due to indirect effect of benzyl propylene glycoside on miR-1976. Previous emerging studies have demonstrated miR-1976 role as a prognostic indicator and tumor suppressor in non-small lung cancer progression [46]. Moreover, it was reported that the miR-1976 knockdown significantly inhibited cell apoptosis and increased cell proliferation [47]. miR-1976 was also found to be one of the specific downregulated exosomal-miRNA signatures related to pancreatic lesions [48]. Interestingly, the functional enrichment analysis of miR-1976 revealed that it is highly linked to inflammatory cGAS-STING-related and fibrogenic pathways including NF-KB signaling, TGF signaling, and TNF signaling pathways.

Herein, DDX58, NFκB1, and CHUK were screened as target genes of miR-1976 using the mirwalk3 database. miR-1976 can regulate the expression level of these genes via binding to their 3ʹUTR resulting in post-transcriptional inhibition or their degradation [47]. Accordingly, the results showed that there was a significant decrease in the expression level of pancreatic miR-1976 in the untreated NAFP group, in comparison with the Sham control. While benzyl propylene glycoside administration significantly increased its expression, compared to the NAFP group.

**Fig. 7:**
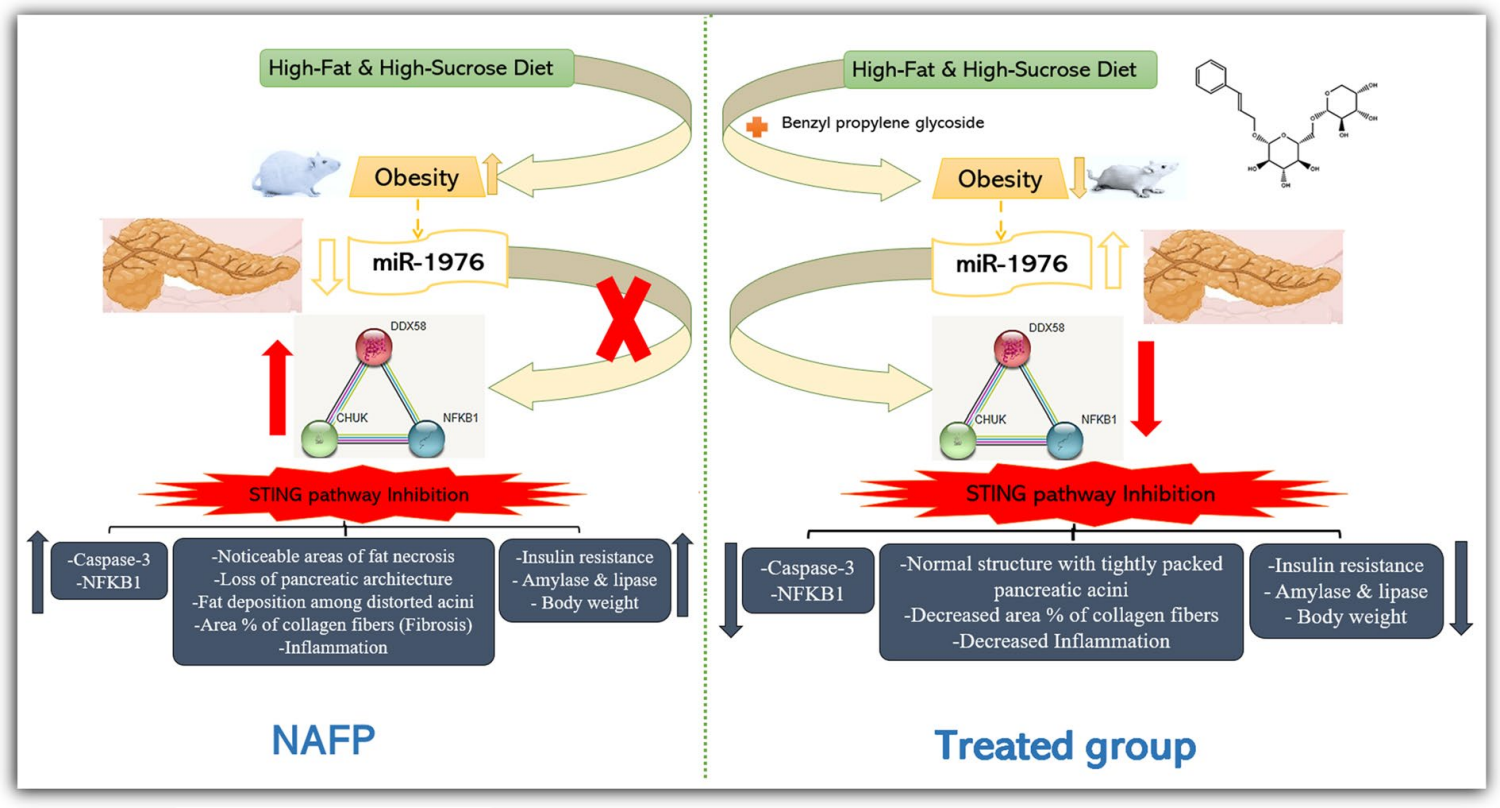
Summary and schematic representation of the study hypothesis. HFHS-induced lipotoxicity (untreated NAFP) downregulated the expression of miR-1976 and thereby upregulated the expression of pancreatic DDX58, NFκB1, and CHUK mRNAs. Activating the cGAS-STING signaling pathway stimulated diverse downstream signaling pathways including promoting the expression of inflammatory and fibrotic responses (NFκB1, Caspase-3, and increase in area percentage of collagen fibers). On benzyl propylene glycoside treatment, increased expression of miR-1976 inhibited the expression of its target genes (DDX58, NFκB1, and CHUK). Inhibiting the cGAS-STING signaling pathway reversed pathological disturbances manifested by decreased inflammation and fibrosis observed in the untreated NAFP. HFHS: high fat and high sucrose diet, NAFP: non-alcoholic fatty pancreas

Taken all together (Fig. 7), we hypothesized that HFHS-induced lipotoxicity (untreated NAFP) downregulated the expression of miR-1976 which could not exert its inhibitory action on its target genes thereby upregulating the expression of pancreatic DDX58, NFκB1, and CHUK mRNAs. Activating the cGAS-STING signaling pathway stimulated diverse downstream signaling pathways, promoted the expression of inflammatory responses (NFκB1 and Caspase-3), increased the area percentage of collagen fibers (fibrosis), and increased the serum level of lipase and amylase. Consequently, increasing pancreatic cell injury and pancreatitis progression. On treatment, the benzyl propylene glycoside increased the expression of miR-1976 and inhibited the expression of its target genes (DDX58, NFκB1, and CHUK). Inhibiting the cGAS-STING signaling pathway reversed metabolic dysfunction, ameliorated insulin resistance, decreased body weight and obesity, and reduced inflammation, and fibrosis observed in the untreated NAFP group.

The present study may help in better understanding the etiology and pathophysiology of the non-alcoholic fatty pancreas disease (NAFP) and also provides useful information regarding potential molecular targets for NAFP treatment. However, benzyl propylene glycoside may not yet be a suitable fundamental mode of therapy until further preclinical trials are performed.

## Conclusion

Benzyl propylene glycoside has demonstrated a potential ability to attenuate NAFP development, inhibit pancreatic cell inflammation and fibrosis and reduce the pathological and metabolic disturbances monitored in the applied NAFP animal model. The detected effect was correlated with upregulation of the expression of pancreatic DDX58, NFκB1, and CHUK mRNAs and downregulation of the expression of pancreatic miR-1976.

## Material and methods

### Drugs and materials

Sodium pentobarbital was obtained from Sigma Aldrich (St. Louis, Missouri, USA). Rosavin (benzyl propylene glycoside) was supplied from Aktin Chemicals, Inc (Cat. #. APC-380, China).

### Animals and treatment

**Fig. 8:**
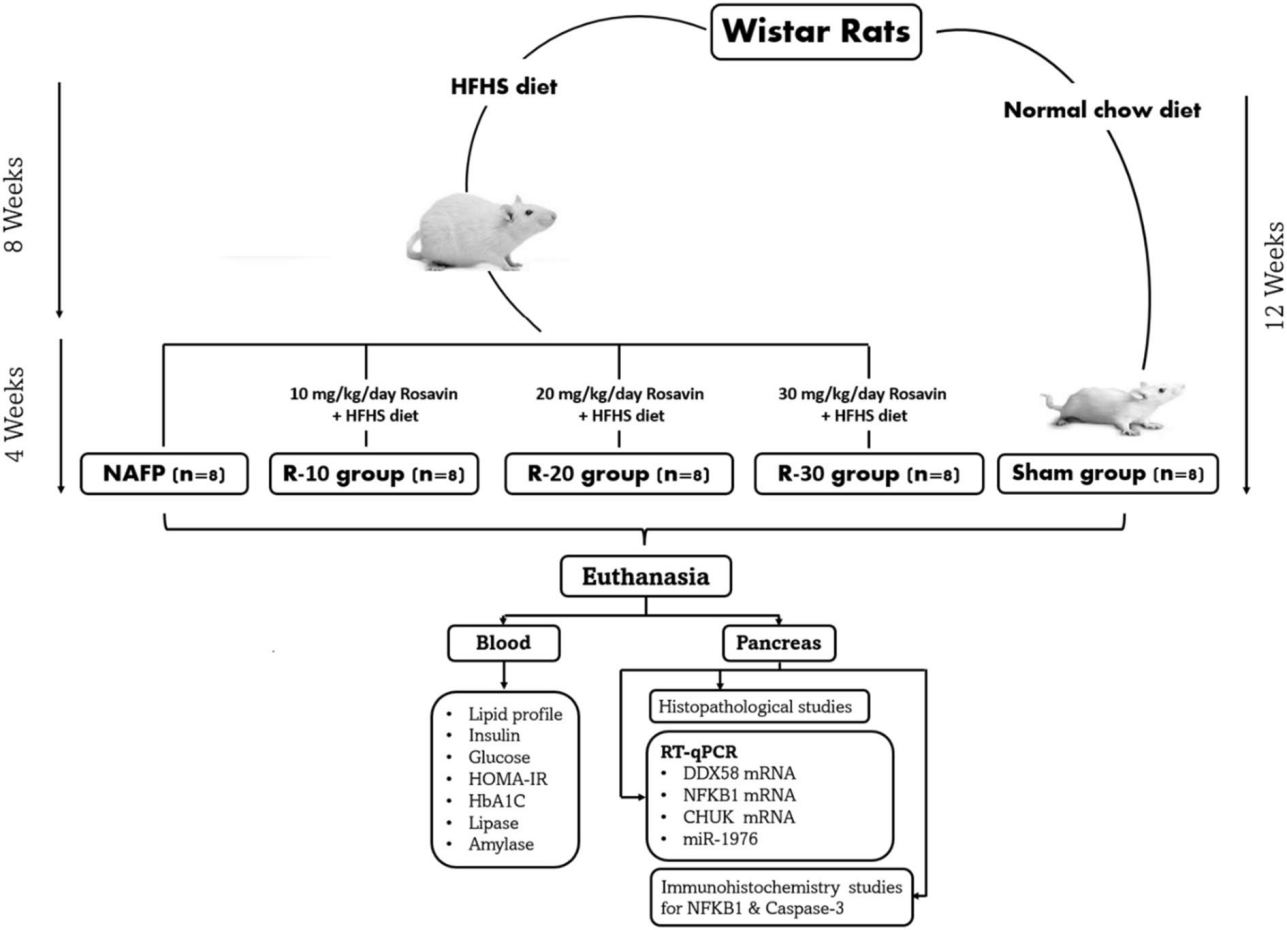
Flowchart showing the experimental design of the study. NAFP: nonalcoholic fatty pancreas; HFHS: high fat and high sucrose

The handling and experimentation protocols were reviewed and approved by the Research Ethics Committee (Number; MoHP0018122017, 1017), Faculty of Medicine, Benha University. The experimental study was performed according to the Declaration of Helsinki guidelines. Male Wistar rats (150–170 g), were housed in cages under standard controlled conditions (12 h light/dark cycles and 21 ± 2 °C) and randomly grouped into normal chow-fed rats (Sham group, n = 8) and high-fat high-sucrose-fed rats (HFHS), Table 4, as a nutritional model for NAFP induction [22]. After 8 weeks of dietary intake, blood samples were drawn to evaluate the effect of the HFHS diet manipulation on the experimental animals. The HFHS-fed animals were then subdivided into 4 groups (n = 8 for each group): untreated HFHS group (NAFP model group) and three benzyl propylene glycoside (rosavin)-treated groups, R-10 group, R-20 group, and R-30 group (Fig. 8). In these treated groups, the rats injected intraperitoneally with10 mg, 20 mg, and 30 mg rosavin/kg body weight, respectively for 4 weeks parallel with HFHS diet [17]. The normal chow-fed rats were given vehicle 0.9% saline intraperitoneally.

**Table 4:** The effect of benzyl propylene glycoside on body weight and blood biochemical parameters

Parameters	Groups					
	Sham	NAFP	HFHS-8 week	R-10	R-20	R-30
Initial body weight, g	160.5 ± 9.40	163.8 ± 10.23	162.7 ± 8.62	164.5 ± 6.83	161.7 ± 7.47	158.2 ± 7.41
Final body weight, g	221.7 ± 9.33	451.8 ± 14.44^*^	353.8 ± 10.23	421.8 ± 42.37	321.3 ± 31.14^###a^	268.2 ± 33.37^###ab^
TC (mg%)	117.3 ± 15.31	273.7 ± 10.57^*^	225.8 ± 8.04^*##^	246.8 ± 9.96^#^	206 ± 8.32^###a^	131.2 ± 20.81^###δab^
TG (mg%)	50.23 ± 5.37	208.4 ± 23.91^*^	143 ± 11.97^*##^	131 ± 27.83^###^	88.6 ± 13.73^###δa^	61.24 ± 5.76^###δa^
HDL-C (mg%)	58.67 ± 4.1	26 ± 4.13^*^	37 ± 2.76^*##^	42 ± 2.48^###^	45.8 ± 1.40^###δ^	50.51 ± 3.89^###δa^
LDL-C (mg%)	44.18 ± 11.55	206 ± 9.13^*^	160.8 ± 7.25^*##^	181.9 ± 10.68^##δ^	137.2 ± 14.57^###δa^	80.11 ± 10.59^###δab^
Glucose (mg%)	101.3 ± 13.9	392 ± 39.01^*^	279 ± 37.77^*##^	260 ± 28.95^###^	151.3 ± 7.94^###δa^	125.6 ± 15.56^###δa^
Insulin (µU/ml)	5.07 ± 1.15	17.78 ± 1.09^*^	15.24 ± 1.28^*#^	14.85 ± 1.09^##^	7.37 ± 1.71^###δa^	6.037 ± 1.26^###δa^
HOMA-IR	1.38 ± 0.27	17.25 ± 2.46^*^	10.75 ± 1.25^*##^	9.547 ± 1.39^###^	2.775 ± 0.75^###δa^	1.85 ± 0.36^###δa^
HbA1C%	4.28 ± 0.94	11.32 ± 1.51^*^	9.883 ± 1.22^*^	8.6 ± 0.74^##^	6.3 ± 0.6^###δa^	5 ± 1.19^###δa^
Lipase (U/L)	350 ± 56.57	3868 ± 166.7^*^	3613 ± 486^*^	3478 ± 511	946.7 ± 133.5^###δa^	595.5 ± 49.73^###δa^
Amylase (U/L)	983.7 ± 111.9	10,927 ± 1273^*^	9245 ± 1504^*^	9185 ± 1611^#^	2931 ± 478.9^###δa^	1190 ± 208.2^###δab^

Values are mean ± SD; number = 8 rats/each group. One-way ANOVA followed by Tukey’s multiple comparison test

^*^p < *0.001* vs Sham

^*###*^*p* < *0.001*

^*##*^*p* < *0.01* and

^*#*^*p* < *0.05* vs NAFP group

^*δ*^*p* < *0.05* vs HFHS-8 week

^*a*^*p* < *0.05* vs R-10

^*b*^*p* < *0.05* vs R-20

### Euthanasia and blood and pancreas tissue collection

All the experimental rats were monitored daily for body weight. At the end of the experimental period (12 weeks), the experimental rats were anesthetized with a single dose of sodium pentobarbital (45 mg/kg, intraperitoneally) [49] and blood samples were rapidly obtained from the retro-orbital vein. Serum was then obtained by centrifugation (1200 g for 10 min) and stored at − 20 °C for the biochemical analyses. The pancreas was carefully removed, weighed, and then rapidly fixed in freshly prepared 10% neutral buffered formaldehyde for analysis by light microscopy.

### Serum biochemical analysis

#### Lipid profile markers and glycated hemoglobin (HbA1C)

Total cholesterol (TC), HDL cholesterol (HDL-C), LDL cholesterol (LDL-C), triglycerides (TG), fasting serum glucose and glycated hemoglobin (HbA1C) were quantitatively determined by the multifunctional biochemistry analyzer (AU680, Beckman Coulter Inc., Brea, CA, USA).

#### Nonalcoholic fatty pancreas (NAFP)-model markers

Serum insulin was measured using a rat sandwich ELISA kit purchased from Invitrogen (Cat. NO. ERINSX10, Waltham, Massachusetts, USA) according to the manufacturer’s instructions. Serum lipase and amylase were measured using commercial kits obtained from Erba Diagnostics (Miami, Florida, USA) according to the protocol supplied with the respective kits. Homeostasis model assessment-insulin resistance (HOMA-IR) was calculated using the following formula: HOMA-IR = [fasting serum insulin (µU/ml) x fasting serum glucose (mg%)]/405 [50].

### Pancreatic histological and immunohistochemistry assays

#### Tissue preparation

The buffered formalin-fixed pancreatic samples were dehydrated using an ascending concentration of alcohol, cleared using methyl benzoate, and mounted in paraffin blocks. Sections were cut at a thickness of 5 μm and stained using hematoxylin and eosin (H&E) and Masson's trichrome stain for the detection of collagen fibers. Other paraffin sections were cut and placed on positively charged slides and were exposed to immune reaction for caspase 3 monoclonal antibody (Cat. No. CPP32 4-1-18, Invitrogen, Waltham, MA, USA) and NFκB1 antibody (Cat. No. BS-3300R, Bioss Antibodies, Woburn, MA, USA). The positive reactions for the caspase 3 and NFκB1 immune-histochemical technique appeared as brown nuclear and cytoplasmic reactions. Negative controls were performed according to the same protocol, but without the usage of the primary antibody. Positive control was performed using a section of tonsils. Finally, the slides were counterstained using Mayer’s hematoxylin. Positive controls were carried out according to the same protocol [51].

#### Morphometric study

The morphometric study was done using an image analyzer Leica Q win V.3 program installed on a computer which connected to a Leica DM2500 microscope (Wetzlar, Germany). Pancreatic slides from all groups were evaluated by morphometric study. Evaluations were obtained from five different slides taken from each rat. Five non-overlapping fields were selected haphazardly and examined for each slide. The pancreatic slides were used to measure:

I-The mean area percentage (%) of collagen fibers in Masson's trichrome stained sections at objective lens X 20.

II- The mean area percentage (%) of positive reaction of caspase-3 and NFκB1 sections (X20).

### Bioinformatics set up

#### Retrieval of the mRNAs-miRNAs panel

**Fig. 9:**
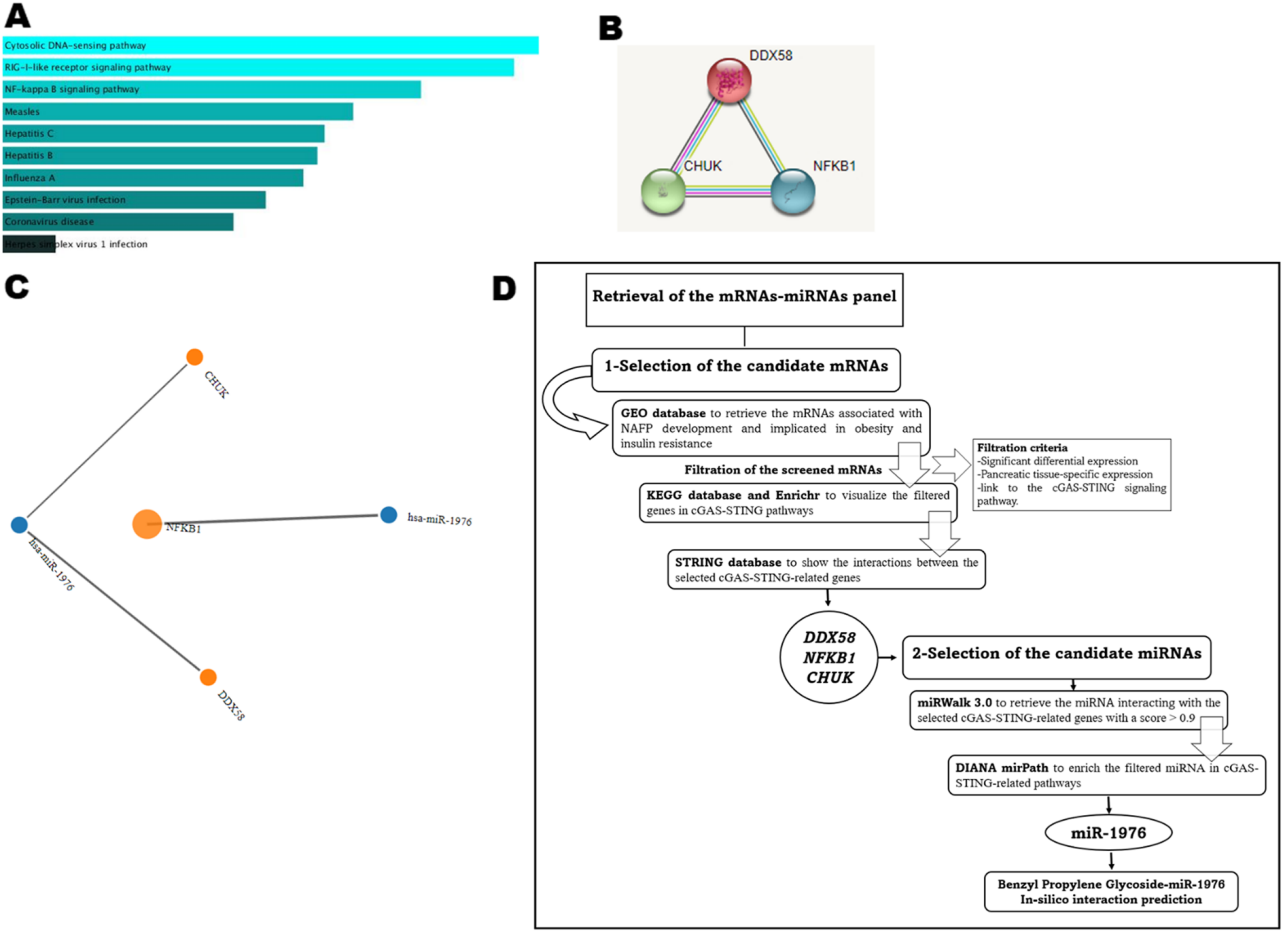
**A** Top 10 items of KEGG pathways for the three selected genes shown in the bar chart according to *p* value obtained with (http://amp.pharm.mssm.edu/Enrichr). **B** The protein–protein interaction (PPI) between the three selected genes using the String tool (http://stringdb.org; version 11.0). **C)** The interaction between the selected genes with the retrieved miR-1976 using miRWalk 3.0 (http://mirwalk.umm.uni-heidelberg.de/). **D** Workflow of bioinformatics Set Up

The RNAs species that are related to NAFP development and implicated in obesity and insulin resistance were searched for. Firstly, the differentially expressed genes (mRNAs) associated with pancreatic injury were screened through the Gene Expression Omnibus (GEO) (www.ncbi.nlm.nih.gov/geo/, accessed on 22 Oct 2021) [52]. The screened mRNAs were further filtered according to their significant differential expression (Additional file 1: Fig. S2), their pancreatic tissue-specific expression (Additional file 1: Fig. S3), and their links to the cGAS-STING signaling pathway. From the filtered mRNAs, DEAD Box Protein 58 (*DDX58*), Nuclear Factor Kappa B Subunit 1 (*NF*κ*B1*), and Conserved Helix-Loop-Helix Ubiquitous Kinase (*CHUK*) were selected as they were validated by other microarray databases (Additional file 1: Fig. S4) and by reviews [53–57] to be related to metabolic syndrome and pancreatic cell dysfunction diseases. The selected genes were also mapped and visualized through the Kyoto Encyclopedia of Genes and Genomes (KEGG) pathway database (https://www.genome.jp/kegg/, accessed on 22 Oct 2021) to be enrolled in the cGAS-STING pathway (Additional file 1: Fig. S5). The pathway enrichment analysis using Enrichr (http://amp.pharm.mssm.edu/Enrichr, accessed on 22 Oct 2021) [58] was primarily enriched in cGAS-STING and NF-kappa B signaling pathways. The top ten terms for pathway enrichment are shown in Fig. 9A. Based on the STRING tool (http://stringdb.org, accessed on 22 Oct 2021) [59], the protein–protein interaction (PPI) between the three selected genes showed a high confidence level with a combined score > 0.7 (Fig. 9B).

Secondly, miRWalk 3.0 (http://mirwalk.umm.uni-heidelberg.de/, accessed on 22 Oct 2021) was utilized for the retrieval of miRNAs interacting with the three selected mRNAs. miR-1976 (Fig. 9C) was found to target the 3 selected mRNAs with a score ˃ 0.9 (Additional file 1: Fig. S6). DIANA tools mirPath (http://www.microrna.gr/miRPathv3, accessed on 22 Oct 2021) was then used to track pathways of miR-1976. Interestingly, miR-1976 was detected to be related to cGAS-STING-related pathways (Additional file 1: Fig. S7).

All in all, the mRNAs (*DDX58*, *NF*κ*B1*& *CHUK*)—(miR-1976) panel was constructed.

#### Molecular docking analysis: benzyl propylene glycoside (Rosavin)—miR-1976 in-silico interaction prediction

The molecular docking between the upstream regulator miR-1976 and rosavin was performed. Rosavin ligand was obtained from PubChem with ID: 9,823,887. The miRNA1976 sequence was extracted from the miRbase database with accession number: MI0009986. The secondary structure was computed using RNAFold under ViennaRNA package (Version 2.4.18) [60]. The minimum free energy (MFE) of the secondary structure was computed at 37 °C. The secondary structure was subjected to 3D modelling using RNAComposer web server [61, 62]. The 3D model was used for docking using HDock software which models the protein using two algorithms: template-based and ab initio modelling [63]. The docked forms are ranked upon their docking scores, Root mean standard deviation and confidence score according to the HDock manual. The predicted Rosavin-miRNA1976 interaction for the top 10 poses were calculated using BIOVIA Drug Discovery Studio Visualizer 2021 (version 21.1.0.20298).

### Total RNA extraction and quantitative polymerase chain reaction (qPCR)

Total RNA, involving mRNAs and miRNAs, extraction from the 60 mg of frozen pancreas tissue samples was performed using a miRNEasy extraction kit (Qiagen, Hilden, Germany, Cat. No. 217004) according to the protocol supplied with the kit. NanoDrop (Thermo scientific, USA) was utilized to assess the concentration and purity of total RNA and the purity of the isolated RNAs was adjusted to be 1.8–2 (A260/A280). The RNA extracted from the pancreas tissues was then reverse transcribed into complementary DNA using miScript II RT (Cat. No. 218161, Qiagen, Germany).

Relative expression of the selected RNAs species in the pancreatic tissue samples was assessed using a Quantitect SYBR Green Master Mix Kit (Qiagen, Germany, Cat. No. 204143) for DDX58, NFκB1, and CHUK mRNAs and miScript SYBR Green PCR Kit (Qiagen, Germany, Cat no. 218073) for miR-1976 miRNA. Real-time (RT)-qPCR was conducted on 7500 Fast System (Applied Biosystems, Foster City, USA). The GAPDH and SNORD72 were used as housekeeping genes. The primers list used herein was obtained from Qiagen, Germany (Additional file 1: Table S1). The relative quantification of RNA expression was calculated using RQ = 2 ^–ΔΔCt^ formula [64].

### Statistical analysis

GraphPad Prism software, version 8.0 (Inc., CA, USA) was utilized to perform the Statistical analyses. The distribution normality of the data was analyzed using the Kolmogorov–Smirnov test. Data are represented as the mean ± standard deviation (SD). Differences among groups were analyzed by one-way analysis of variance (ANOVA) for statistical significance, followed by Tukeyʼs test.

## Supplementary Information

**Additional file 1: Figures S1.** The docking poses of Rosavin-miRNA1976 interaction. **Figures S2.** The significant differential expression of the selected candidate genes (*DDX58*, *NF*κ*B1*, and *CHUK)* in pancreatic injury using the Expression Atlas database. **Figures S3.** Validation of the significant expression of the candidate genes/proteins (DDX58, NFκB1, and CHUK) in the pancreatic tissue. **Figure S4.** Validation of the implication of *DDX58*, *NF*κ*B1*, and *CHUK* in metabolic syndrome and pancreatic cell dysfunction diseases. **Figure S5.** The visualization of the selected *DDX58*, *NF*κ*B1*, and *CHUK* genes in the cGAS-STING pathway through KEGG pathway database. **Figure S6.** Validation of the interaction between the selected m-RNAs and the retrieved miR-1976 from mirwalk3. **Figure S7.** Validation of the relation of miR-1976 to cGAS-STING-related pathways through DIANA tools mirPath 3. **Table S1.** List of primer assays.

## Data Availability

All data generated during this study are included in this article.

## Abbreviations

cGAS: Cyclic GMP-AMP synthase
*CHUK*: Conserved helix-loop-helix ubiquitous kinase
*DDX58*: DEAD box protein 58
HFHS: High-fat high-sucrose-fed rats
IR: Insulin resistance
IRFs: Interferons
NAFLD: Non-alcoholic fatty liver disease
NAFP: Non-alcoholic fatty pancreas
NF-kB: Nuclear factor kappa beta
*NF*κ*B1*: Nuclear factor kappa b subunit 1
STING: Stimulator of interferon genes
