# Supplementary material for: Prospective insight into the role of benzyl propylene glycoside as a modulator of the cGAS-STING signaling pathway in the management of nonalcoholic fatty pancreas animal model
Source: Biol Res. 2023 Mar 13;56:11. doi: 10.1186/s40659-023-00423-8 (PMC10010022; doi:10.1186/s40659-023-00423-8)
Supplement: Supplementary file 1 — Additional file 1: Figures S1. The docking poses of Rosavin-miRNA1976 interaction. Figures S2. The significant differential expression of the selected candidate genes (DDX58, NFκB1, and CHUK) in pancreatic injury using the Expression Atlas database. Figures S3. Validation of the significant expression of the candidate genes/proteins (DDX58, NFκB1, and CHUK) in the pancreatic tissue. Figure S4. Validation of the implication of DDX58, NFκB1, and CHUK in metabolic syndrome and pancreatic cell dysfunction diseases. Figure S5. The visualization of the selected DDX58, NFκB1, and CHUK genes in the cGAS-STING pathway through KEGG pathway database. Figure S6. Validation of the interaction between the selected m-RNAs and the retrieved miR-1976 from mirwalk3. Figure S7. Validation of the relation of miR-1976 to cGAS-STING-related pathways through DIANA tools mirPath 3. Table S1. List of primer assays. [file 40659_2023_423_MOESM1_ESM.docx]

Prospective Insight into the Role of Benzyl Propylene Glycoside as a Modulator of the cGAS-STING Signaling Pathway in the Management of Nonalcoholic Fatty Pancreas Animal Model

Journal: Biological Research

**Reda Albadawy ^1,*^, Amany Helmy Hasanin ^2^,** **Sara H. A. Agwa ^3^, Shaimaa Hamady ^4^,**  **Reham Hussein Mohamed ^2^,** **Eman Gomaa^5^, Mohamed** **Othman ^6^,** **Yahia A. Yahia^7, 8^,** **Amani Mohamed Abdel Ghani^9^, Marwa Matboli ^10,*^**

^1^ Department of Gastroenterology, Hepatology & Infectious Disease, Faculty of Medicine, Benha University, Benha 13518, Egypt

^2^ Clinical pharmacology department, Faculty of medicine, Ain Shams University, Cairo, Egypt

^3^ Clinical Pathology and Molecular Genomics Unit, Medical Ain Shams Research Institute (MASRI), Faculty of Medicine, Ain Shams University, Cairo 11382, Egypt

^4^ Department of Biochemistry, Faculty of Science, Ain Shams University, Cairo 11566, Egypt

^5^ Histology and Cell biology department, Faculty of Medicine, Ain Shams University, Giza, Egypt

^6^ Gastroenterology and Hepatology Section, Baylor College of Medicine, Houston, TX 77030, USA

^7^ Chemistry Department, School of Science and Engineering, American University in Cairo, New Cairo 11835, Egypt

^8^ Biochemistry Department, Faculty of Pharmaceutical Sciences and Drug Manufacturing, Misr University for Science and Technology, Giza, Egypt

^9^ Clinical Pathology, Faculty of Medicine, Ain Shams University, Cairo 11566, Egypt; dr_amani83@med.asu.edu.eg

^10^ Medical biochemistry and molecular biology department, Faculty of medicine, Ain Shams University, Cairo 11566, Egypt

* Correspondence: [DrMarwa_Matboly@med.asu.edu.eg](mailto:DrMarwa_Matboly@med.asu.edu.eg) (M.M.) & [reda.albadawy@fmed.bu.edu.eg](mailto:reda.albadawy@fmed.bu.edu.eg)

**Supplementary information:**

**S1 figures:** The docking poses of Rosavin-miRNA1976 interaction: are sketched as 2D diagram with interactions between nucleotides and Rosavin where the types of interacting bonds are as follow: Green interaction: Hydrogen bonding. Cyan interaction: Hydrogen doner bond. Pink interaction: T-shaped pi-pi interaction. Orange interaction: pi-pi anionic interaction.

The length of the bond interaction are reported in Angstrom.

**Model 2:**

**
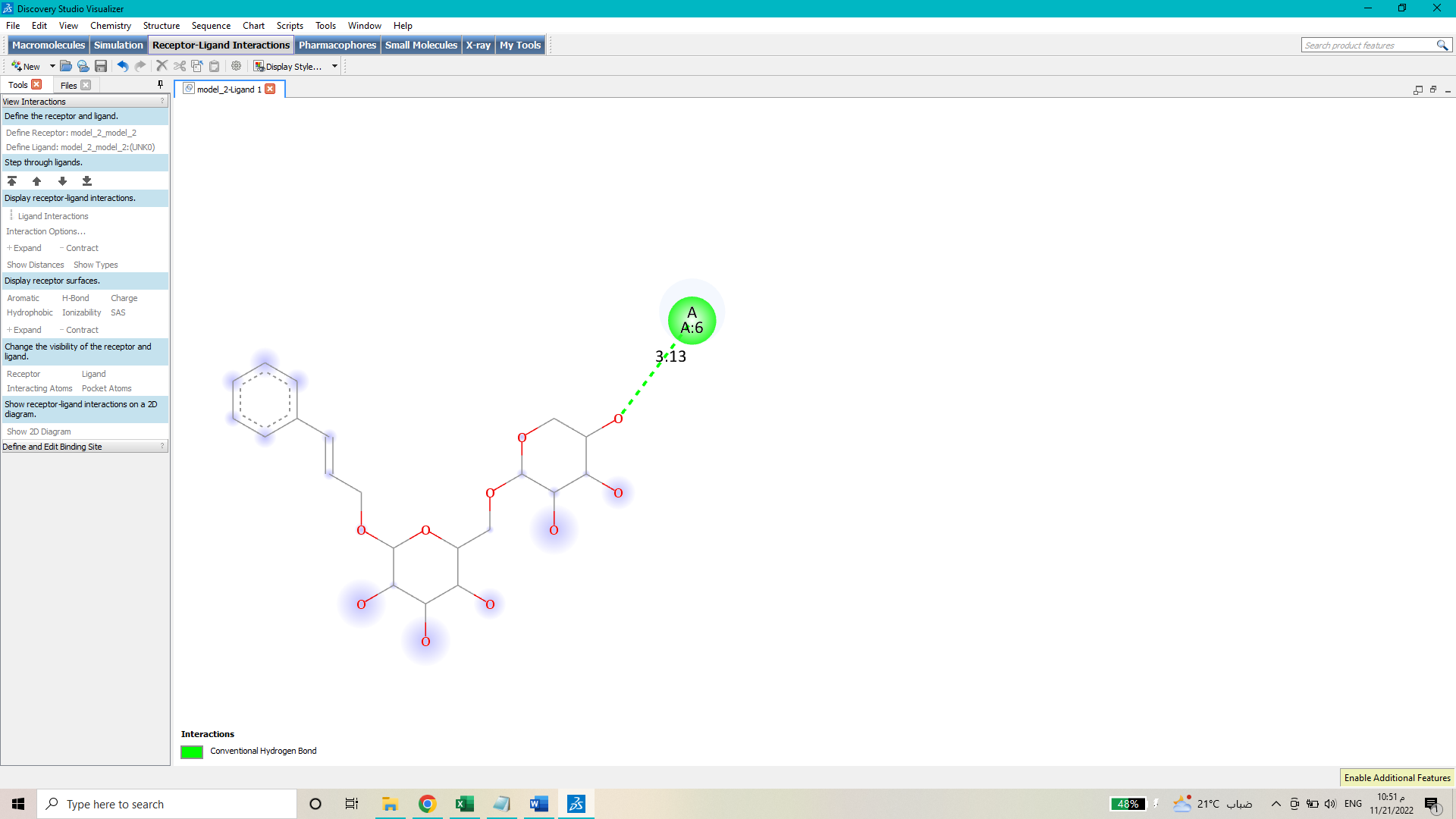
**

**Model 3:**

**
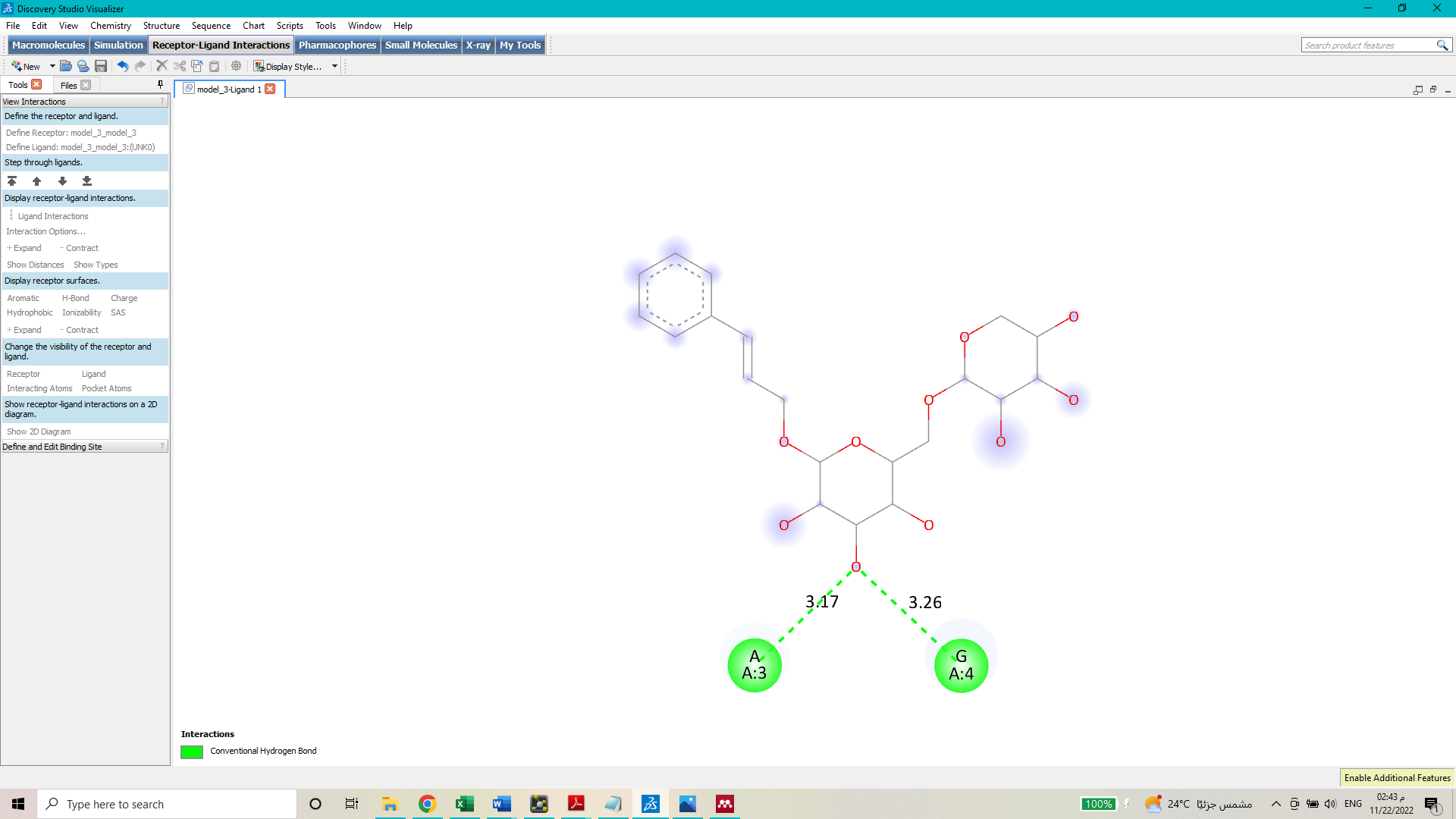
**

**Model 4:**

**
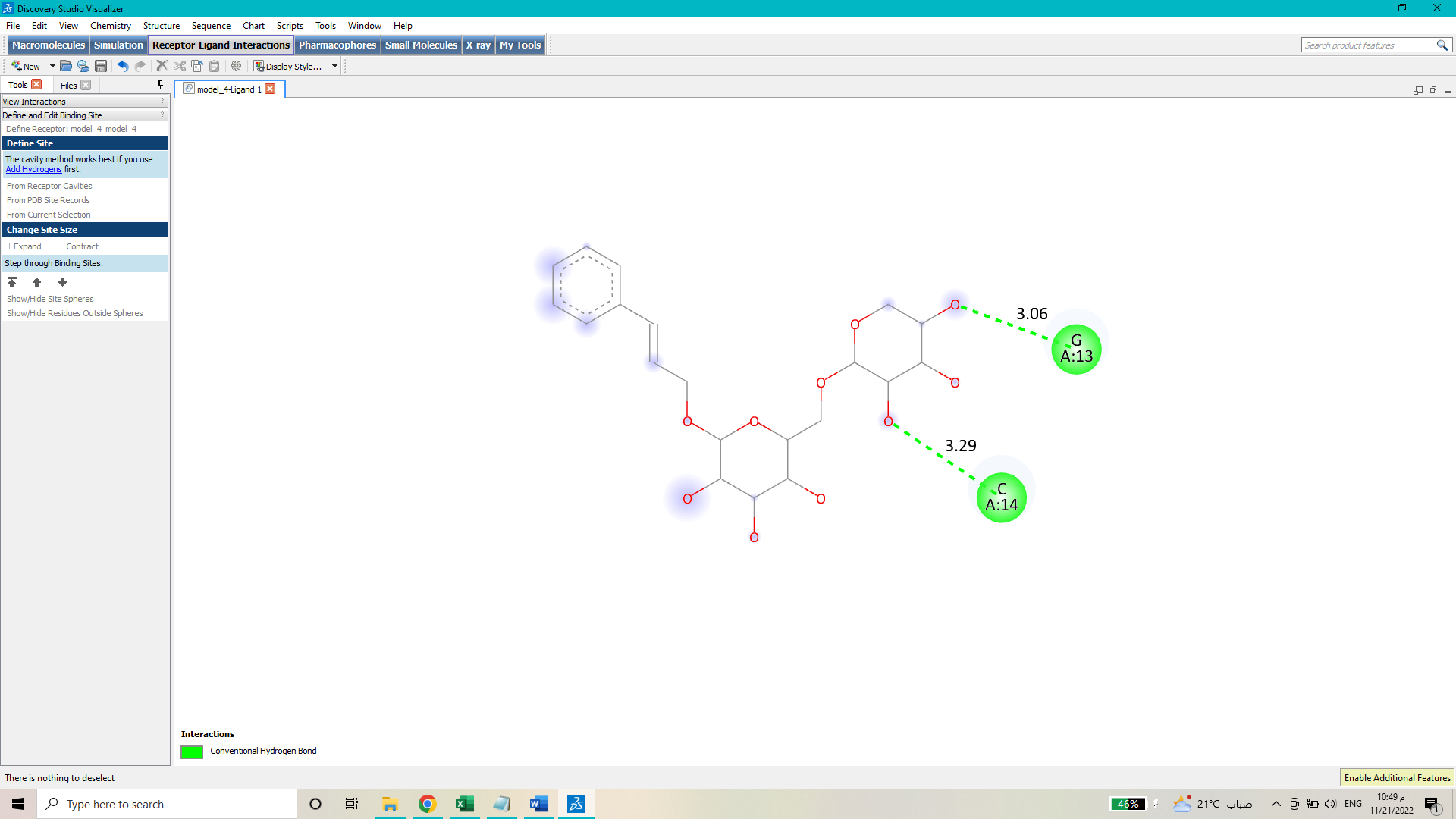
**

**Model 5:**

**
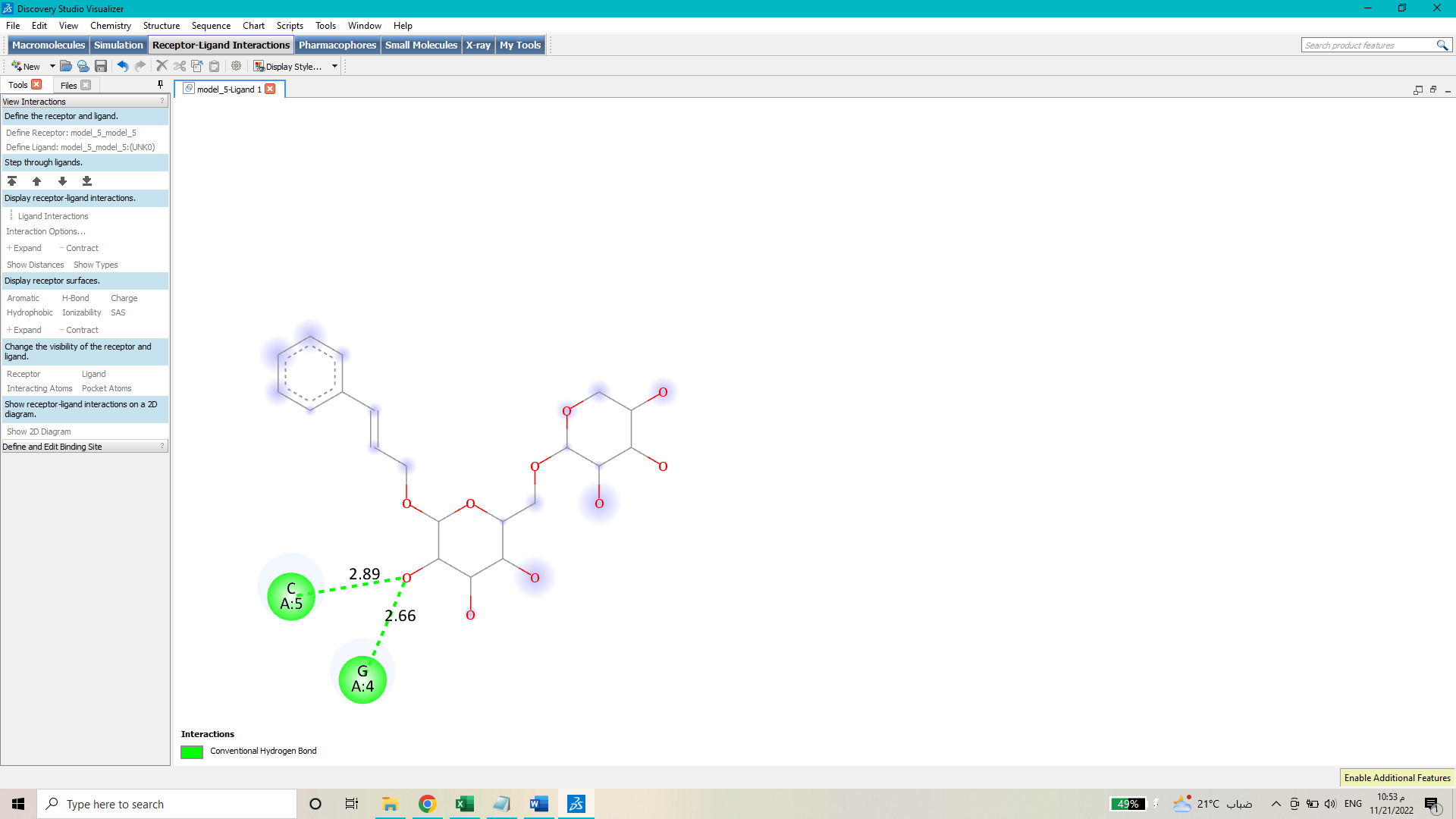
**

**Model 6:**

**
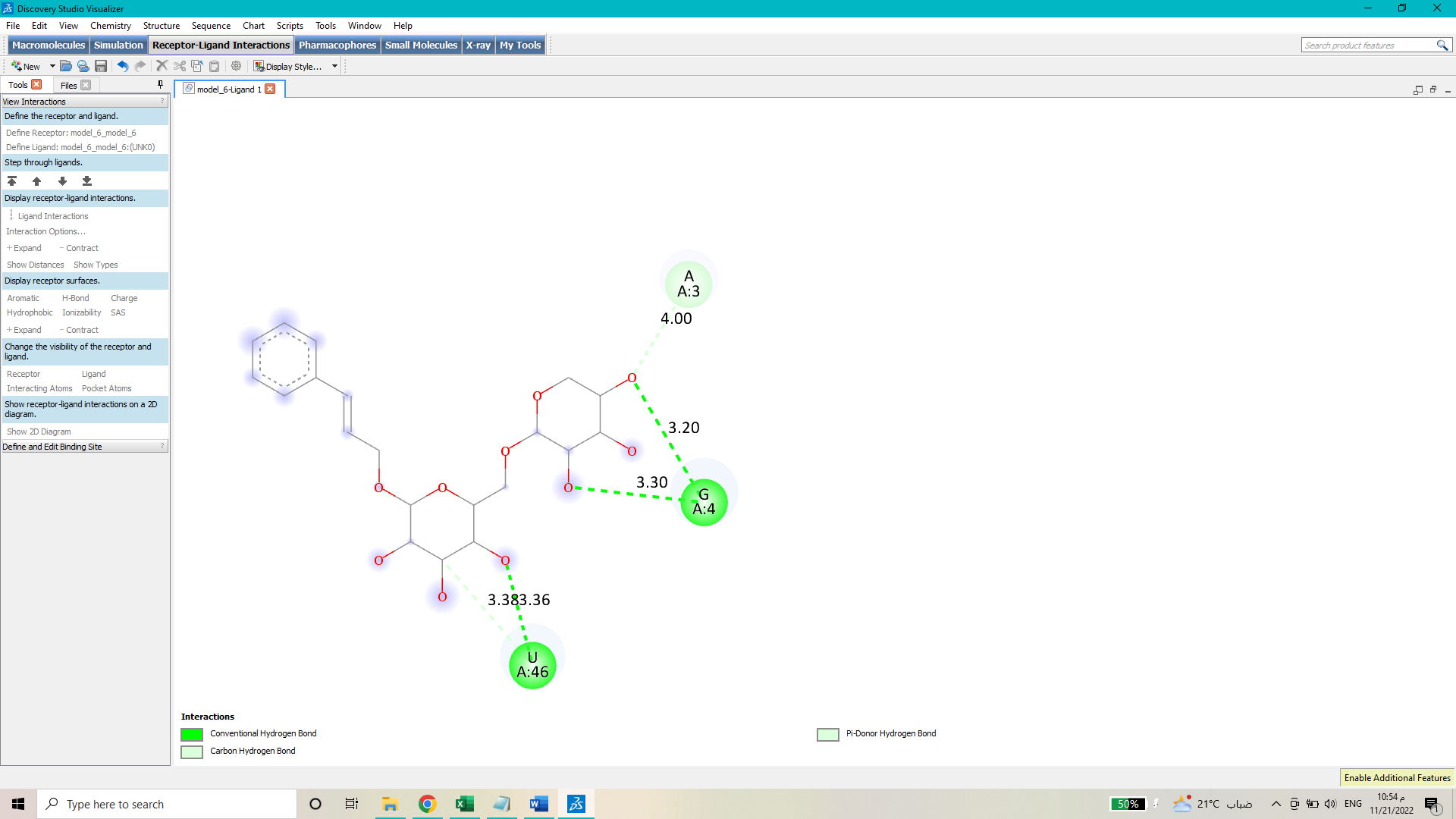
**

**Model 7:**

**
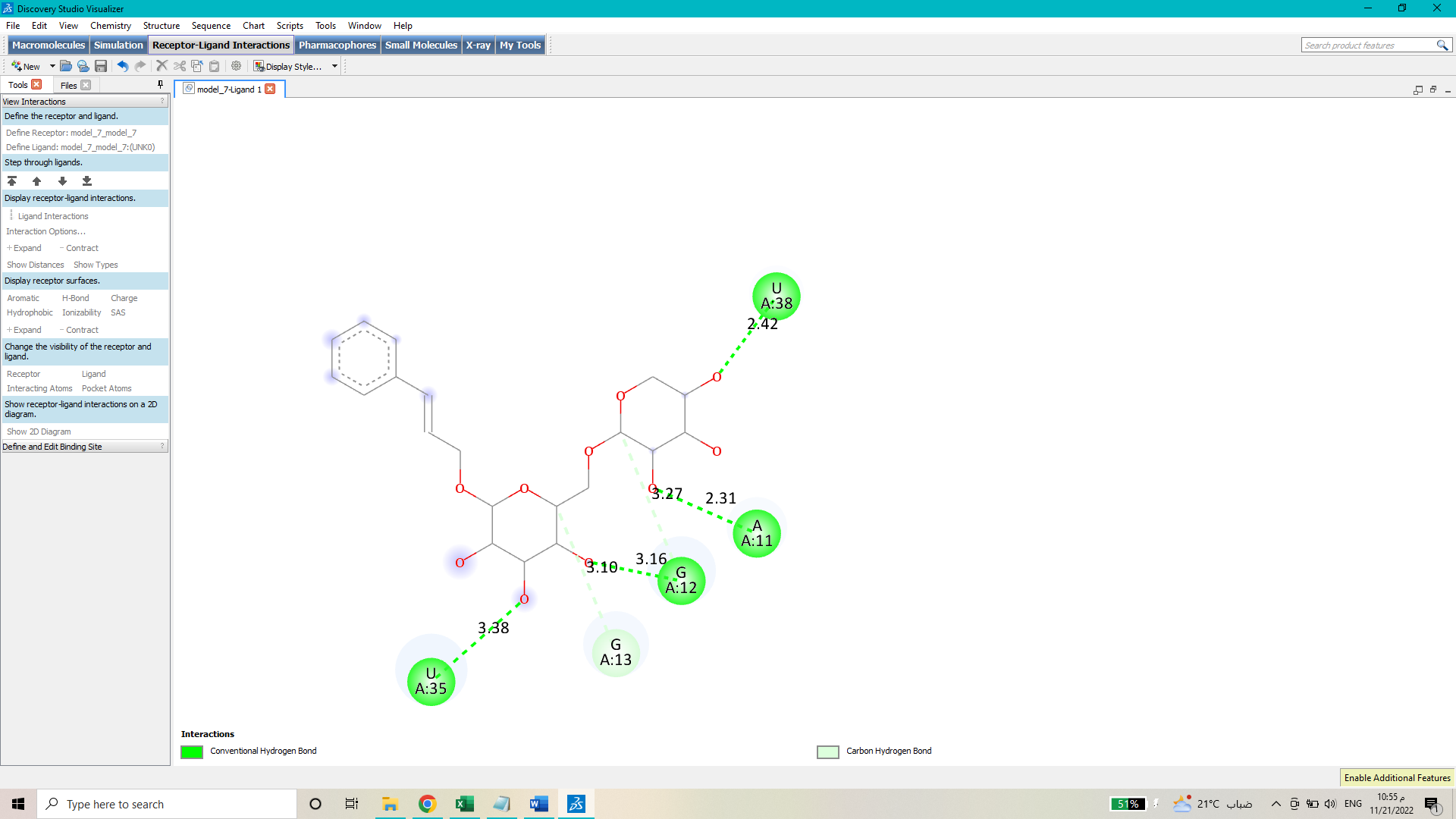
**

**Model 8:**

**
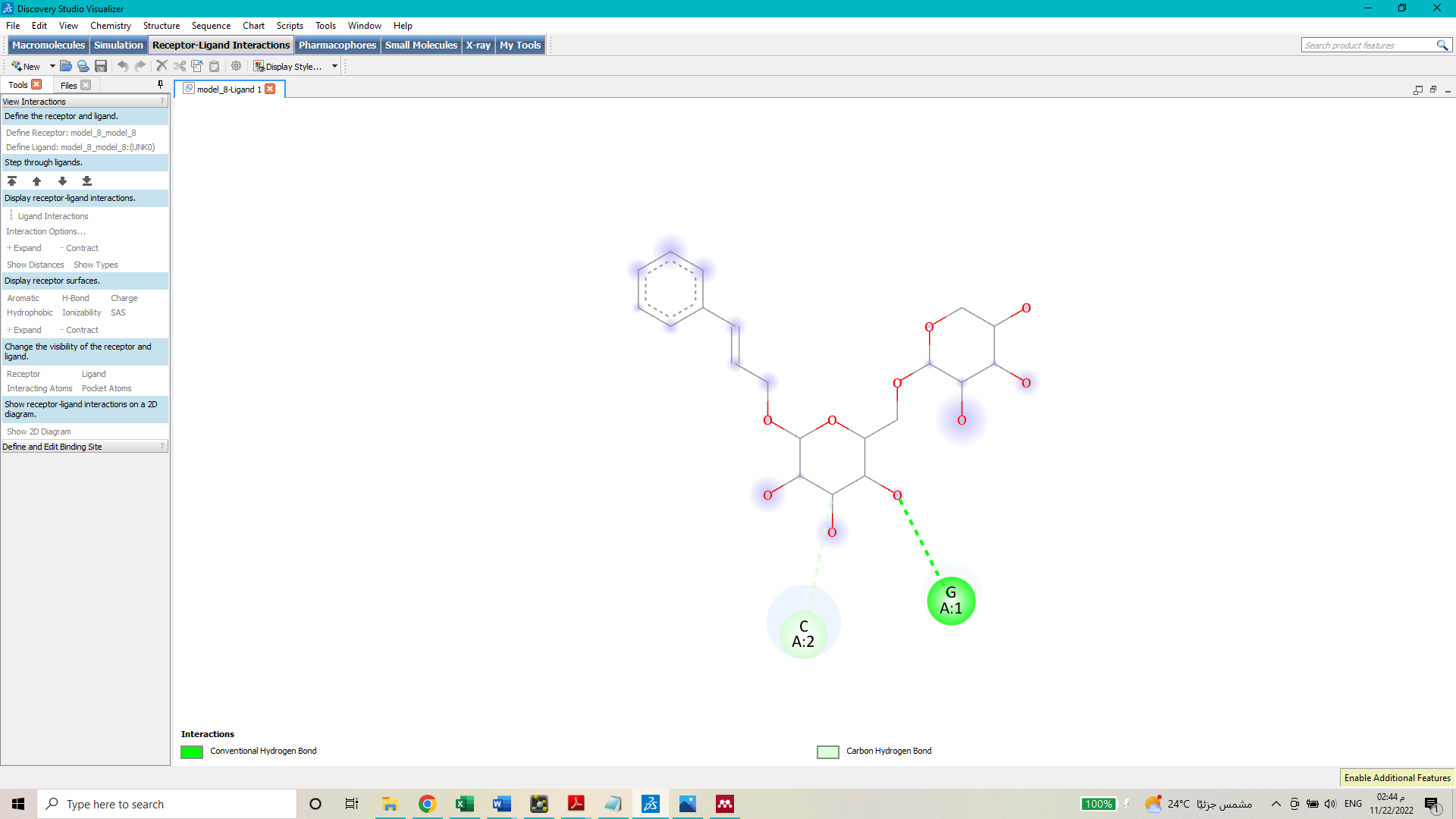
**

**Model 9:**

**
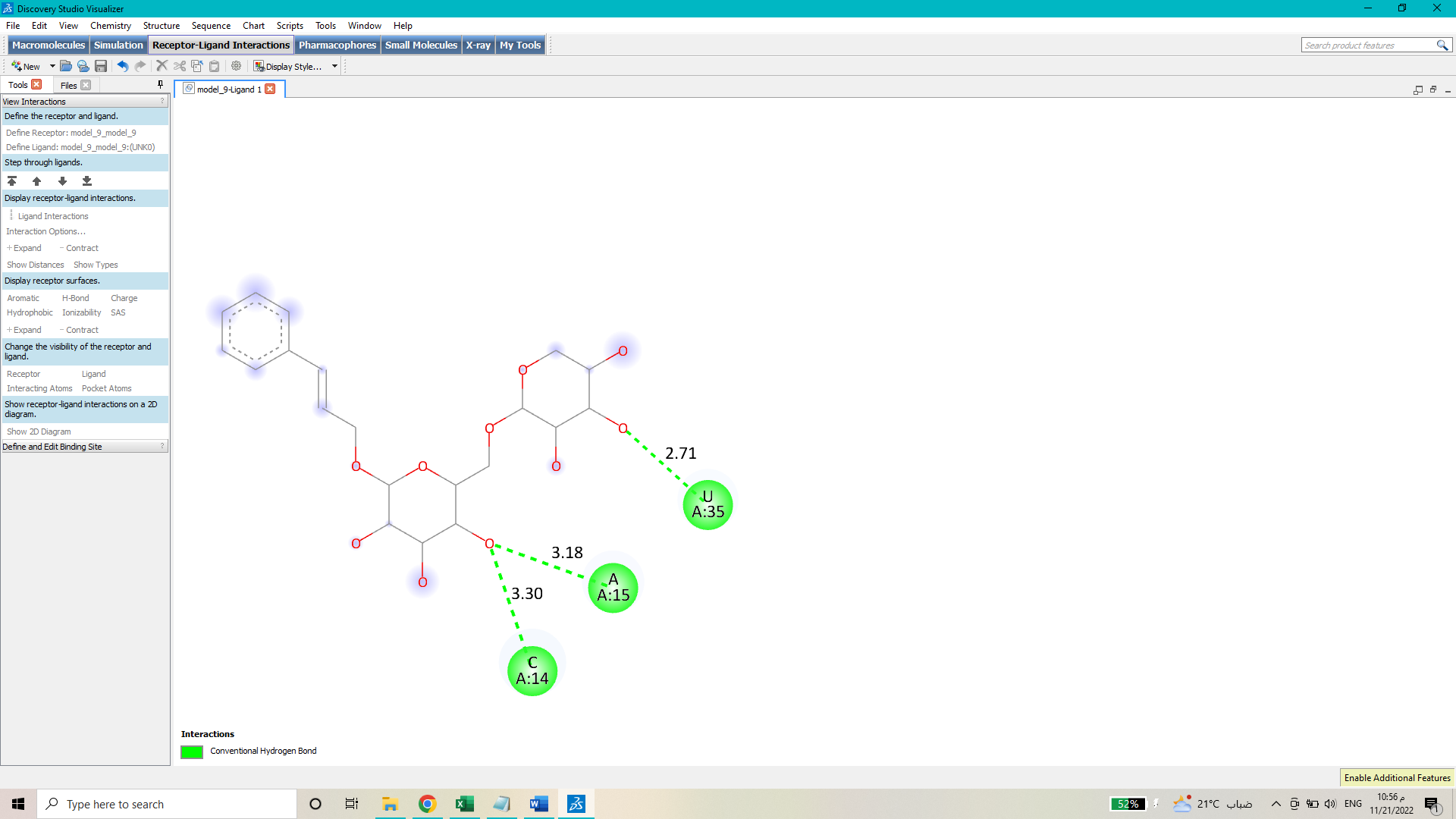
**

**Model 10:**

**
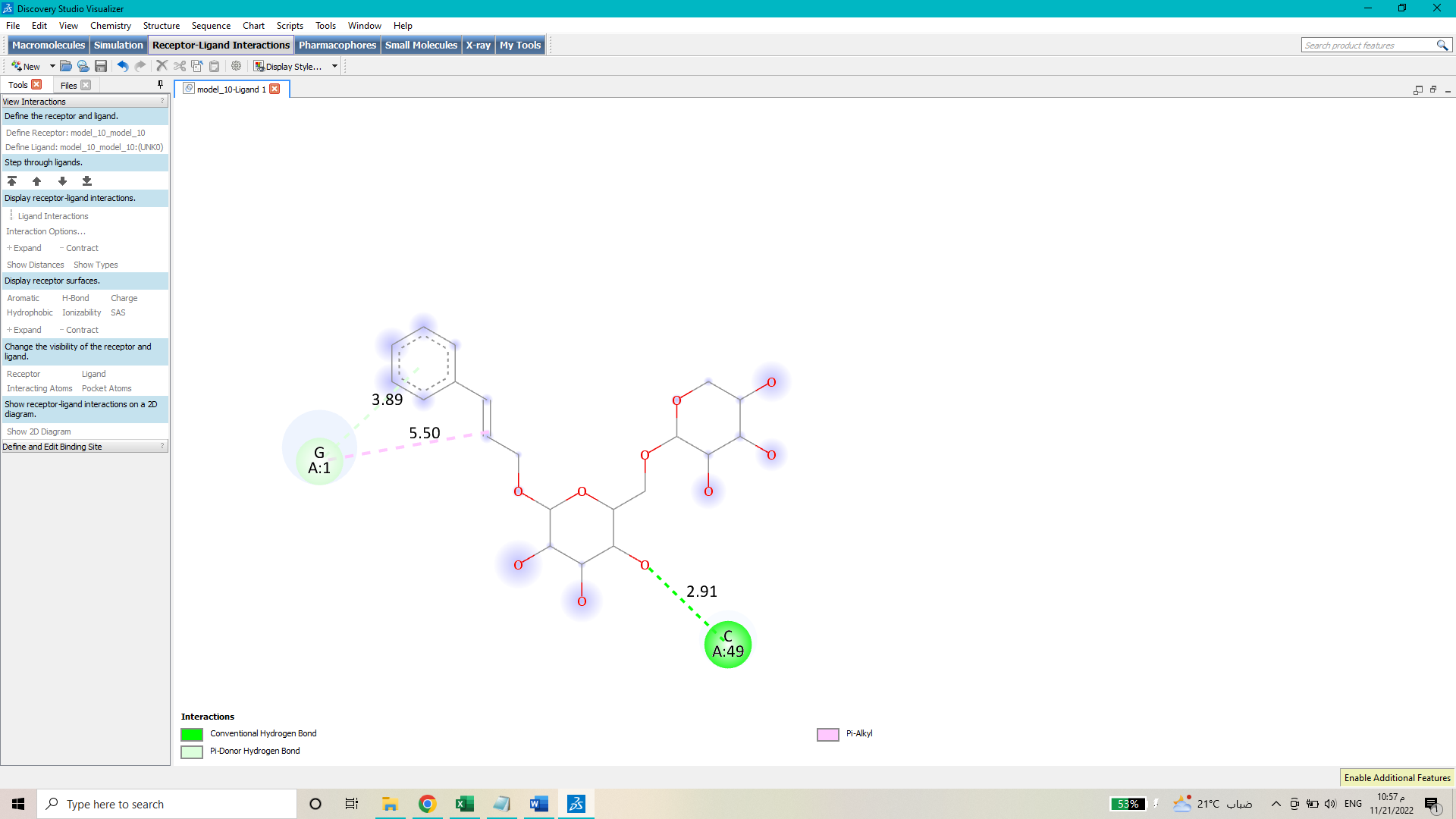
**

**S2 Figures:** The significant differential expression of the selected candidate genes (*DDX58*, *NFKB1*, and *CHUK)* in pancreatic injury using the Expression Atlas database <https://www.ebi.ac.uk/gxa/home>, accessed on 22 Oct 2021

**
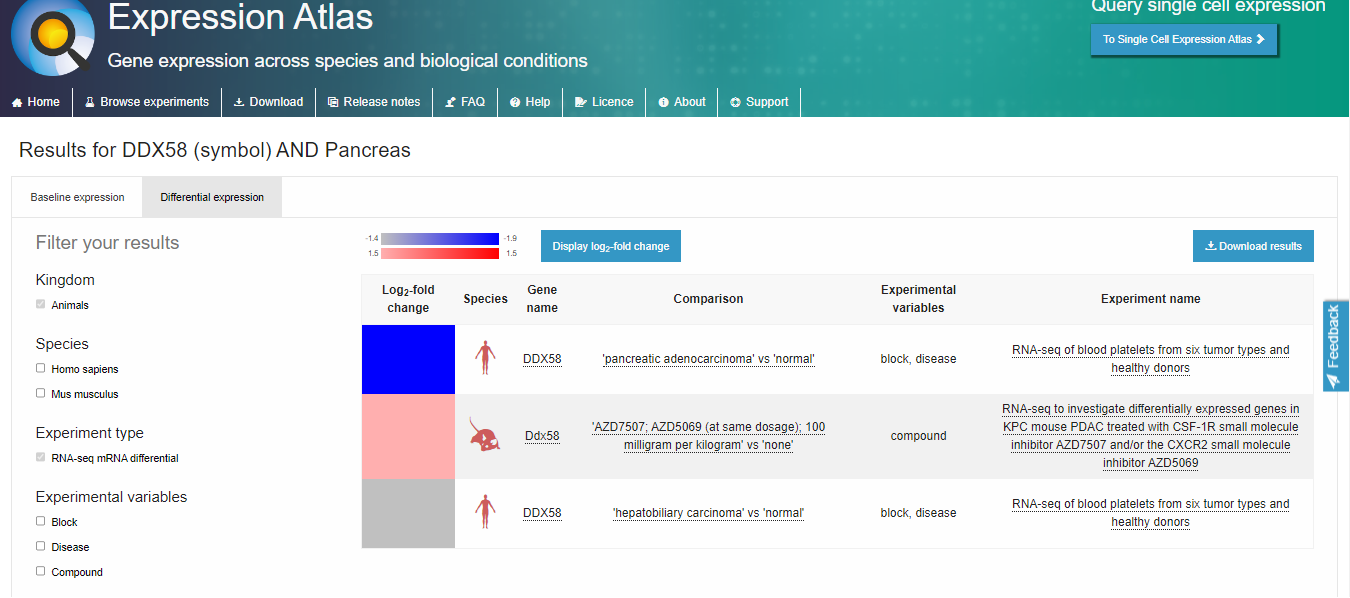
**

**
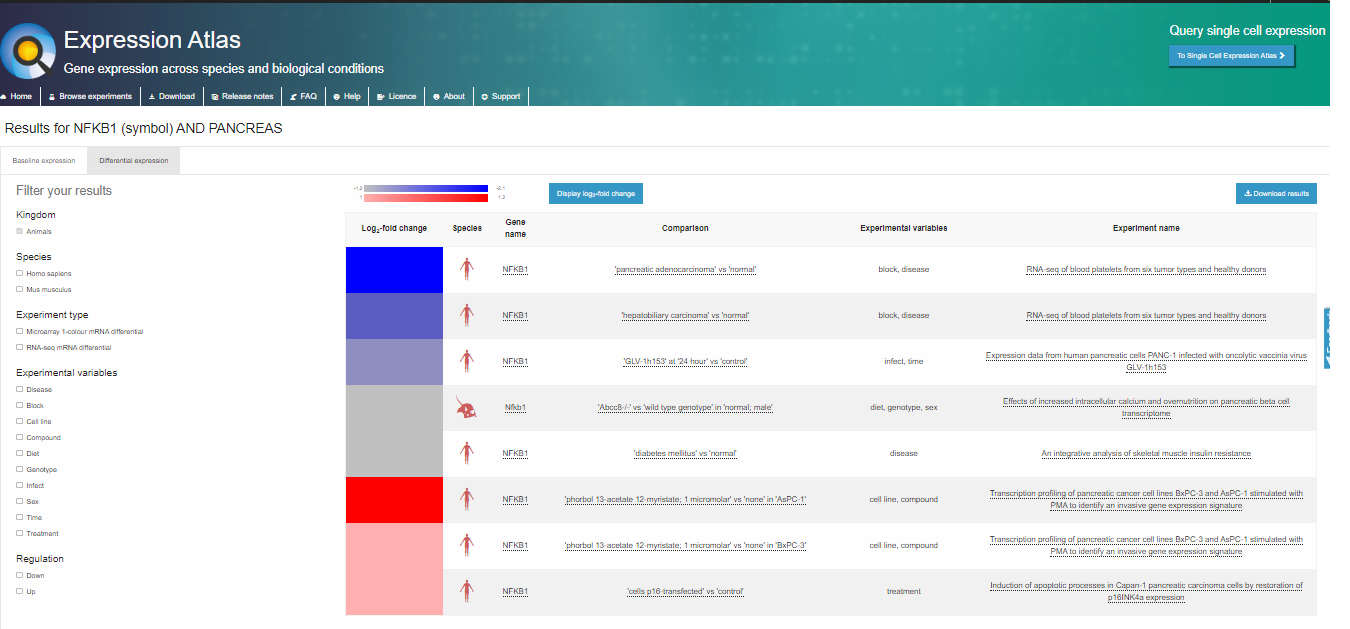
**

**
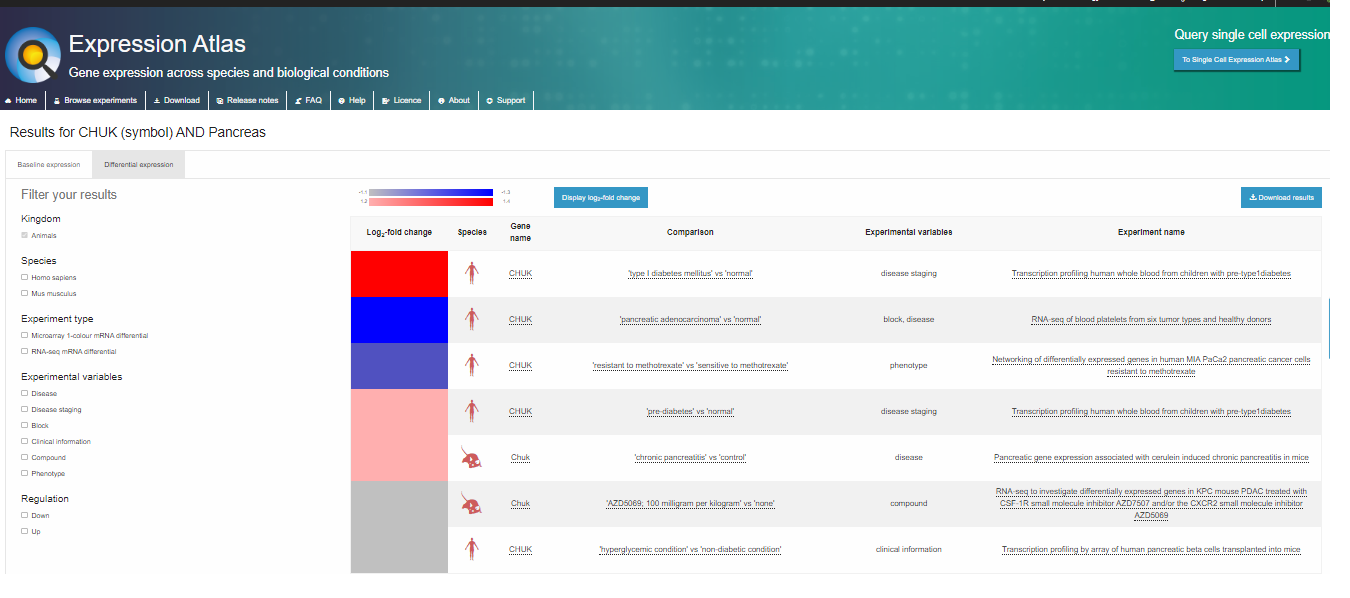
**

**S3 Figures:** Validation of the significant expression of the candidate genes/proteins (DDX58, NFKB1, and CHUK) in the pancreatic tissue using THE HUMAN PROTEIN ATLAS database <https://www.proteinatlas.org/>, accessed on 22 Oct 2021

**
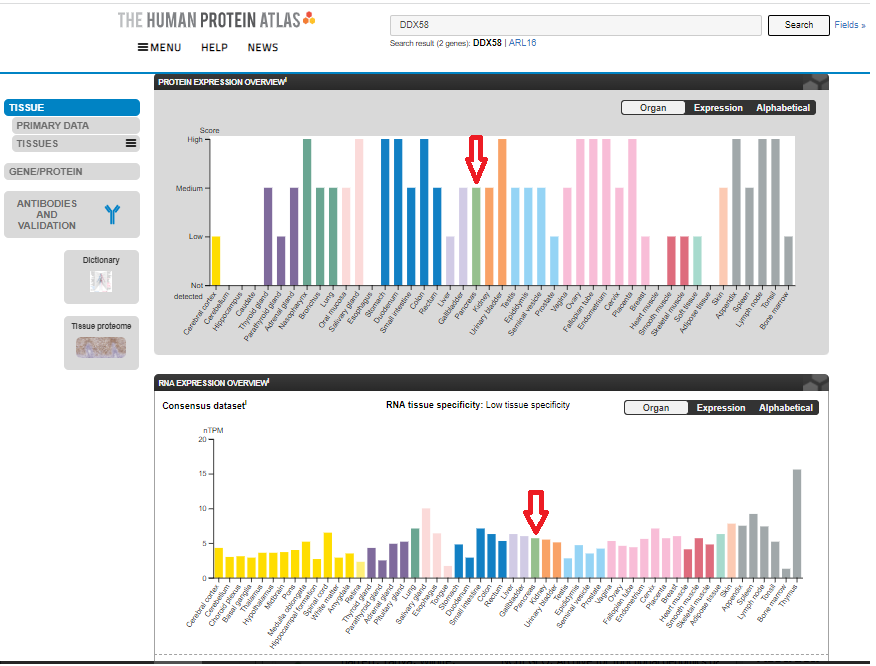
**

**
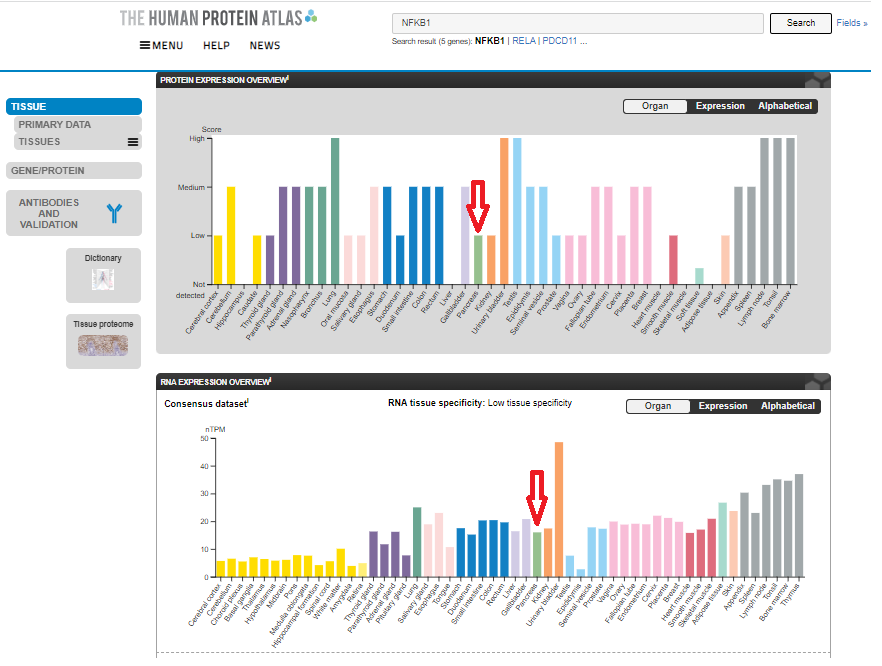
**

**
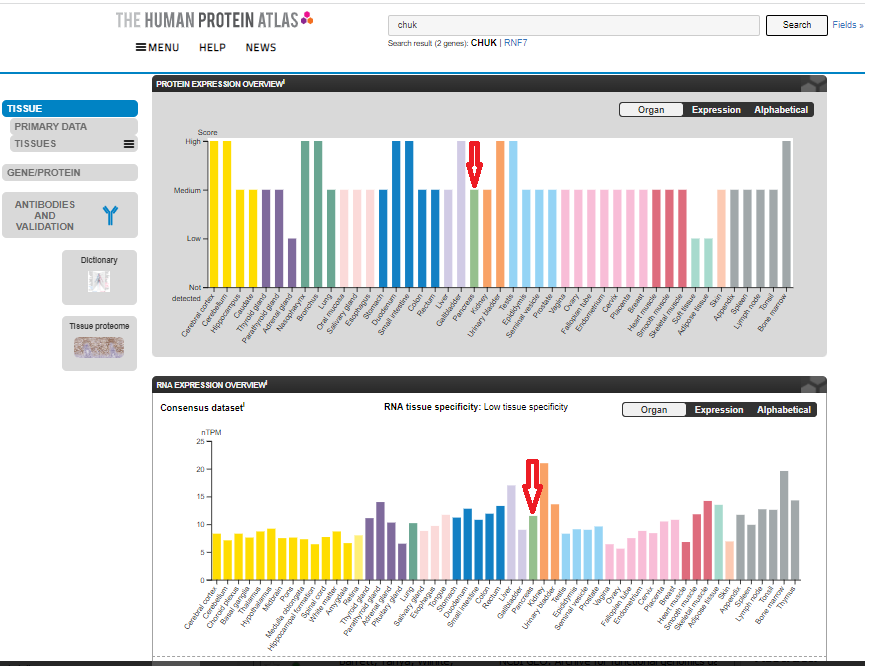
**

**S4 Figure:** Validation of the implication of *DDX58*, *NFKB1*, and *CHUK* in metabolic syndrome and pancreatic cell dysfunction diseases using DisGeNET database (<https://www.disgenet.org/home/>, accessed on 22 Oct 2021)

**
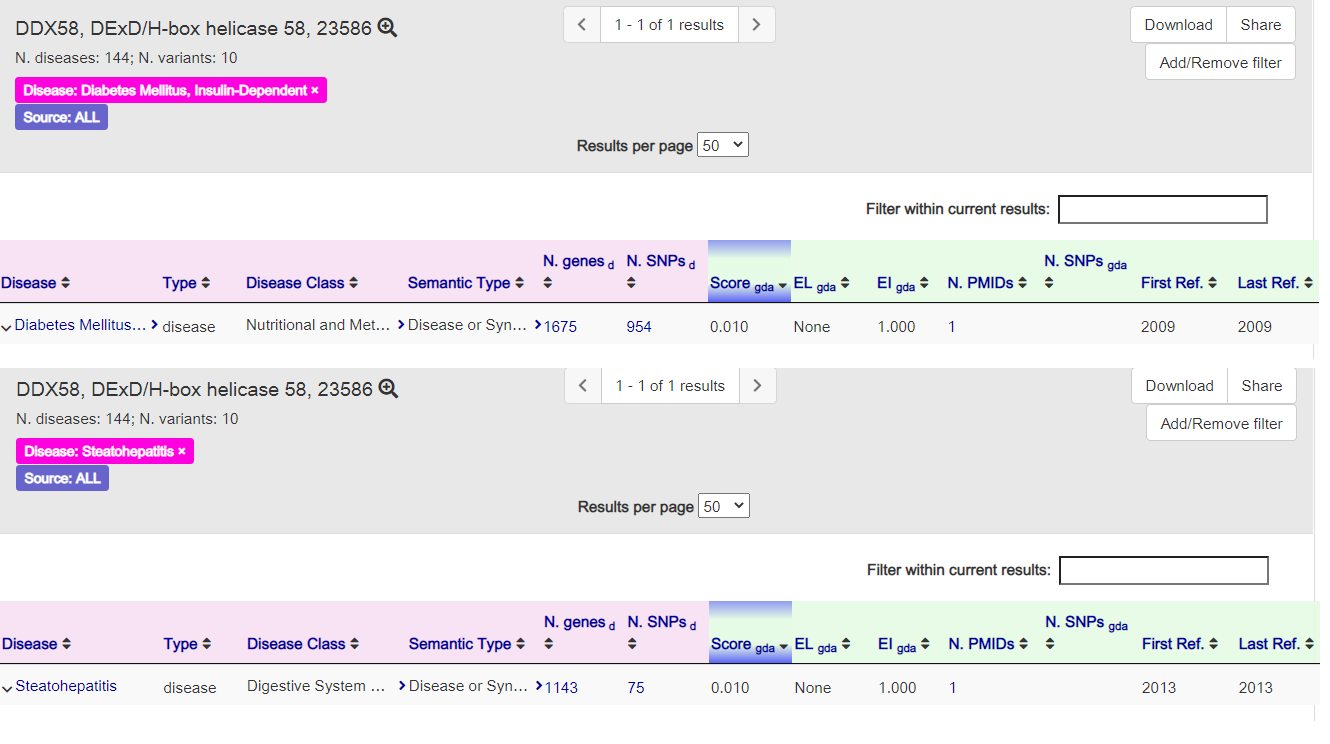
**

**
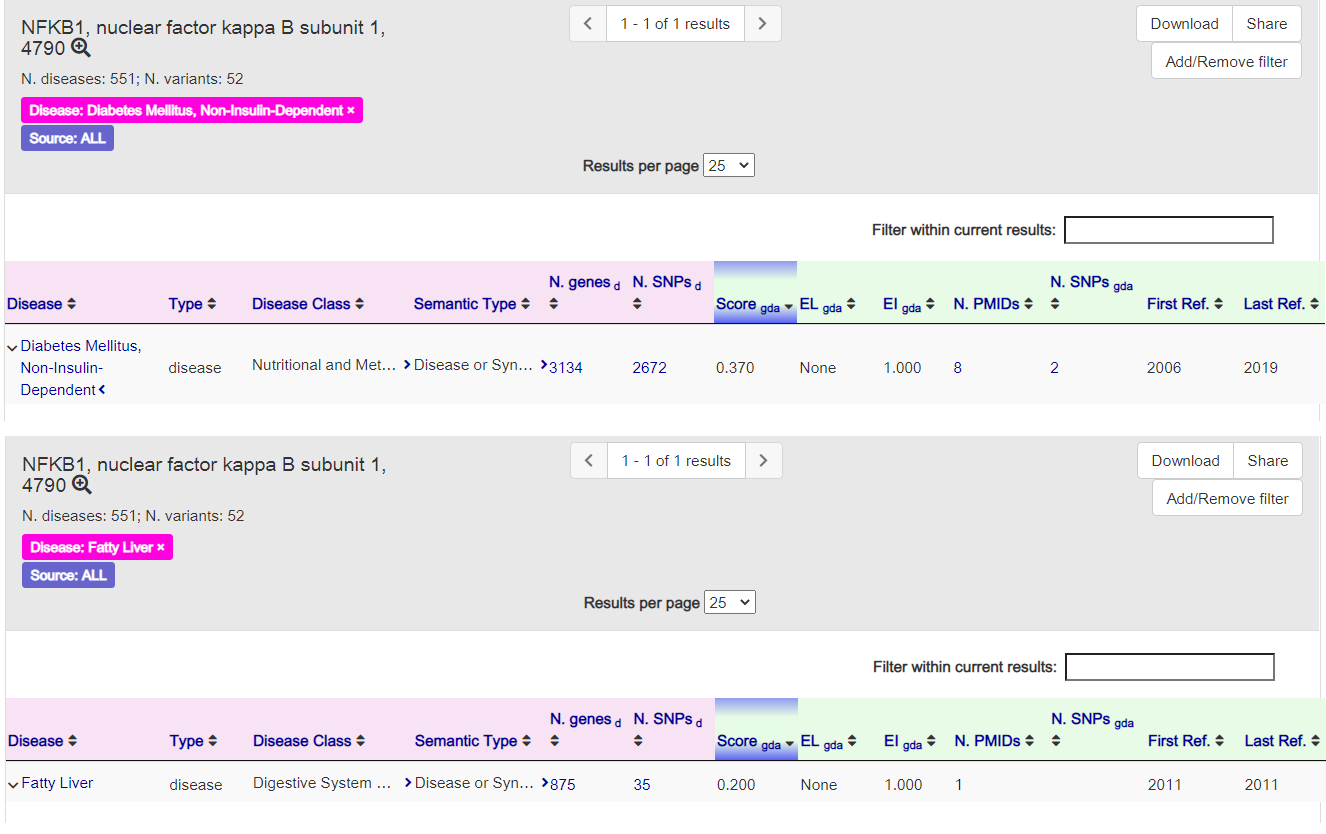
**


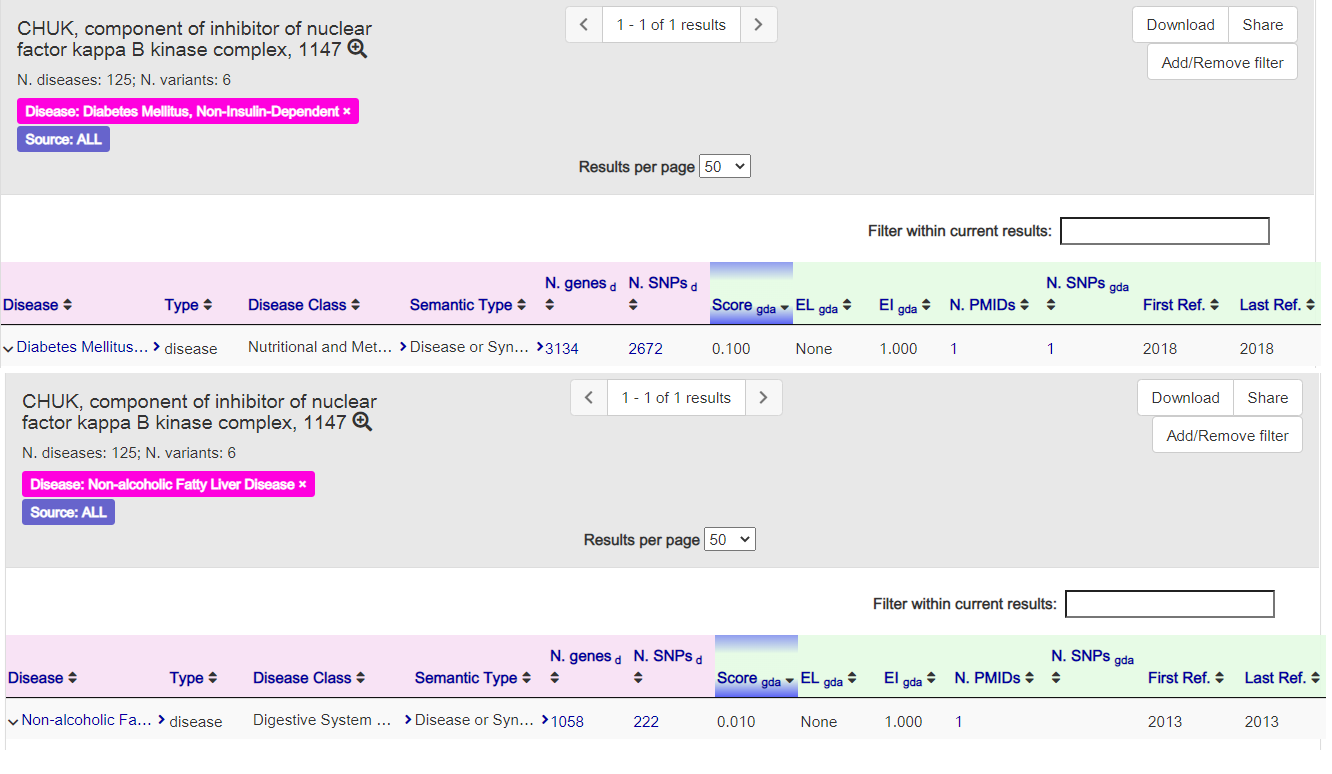


**S5 Figure:** The visualization of the selected *DDX58*, *NFKB1*, and *CHUK* genes in the cGAS-STING pathway through KEGG pathway database (<https://www.genome.jp/kegg/>, accessed on 22 Oct 2021)

**
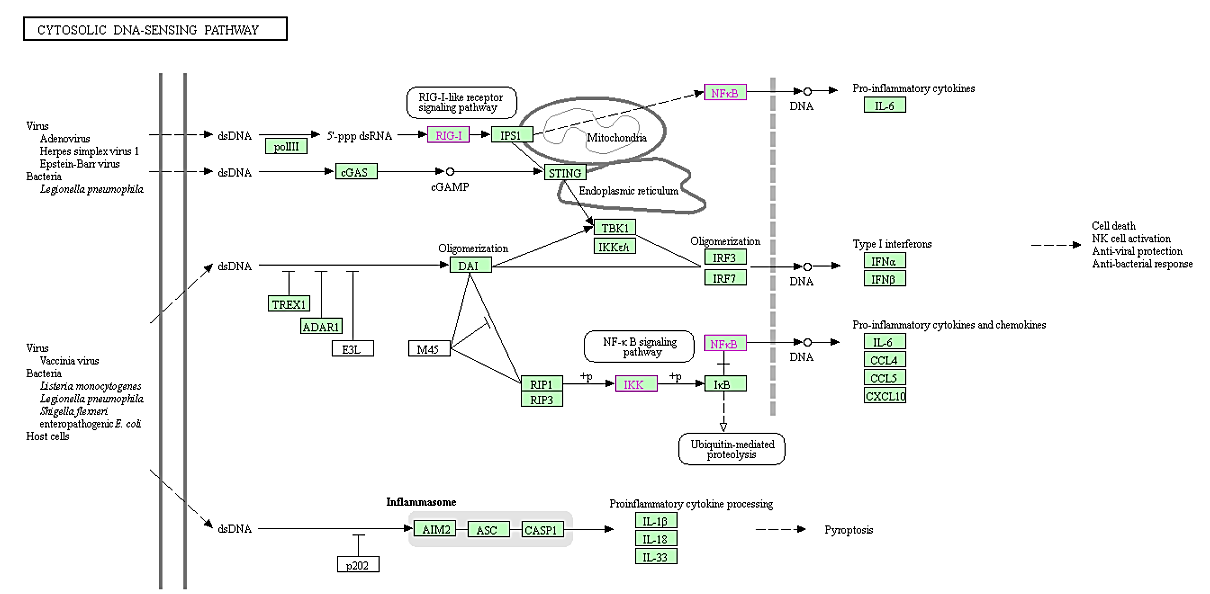
**

RIG-I: DDX58

IKK: CHUK

**S6 Figure:** Validation of the interaction between the selected m-RNAs and the retrieved miR-1976 from mirwalk3 (<http://mirwalk.umm.uni-heidelberg.de/>, accessed on 22 Oct 2021)

**
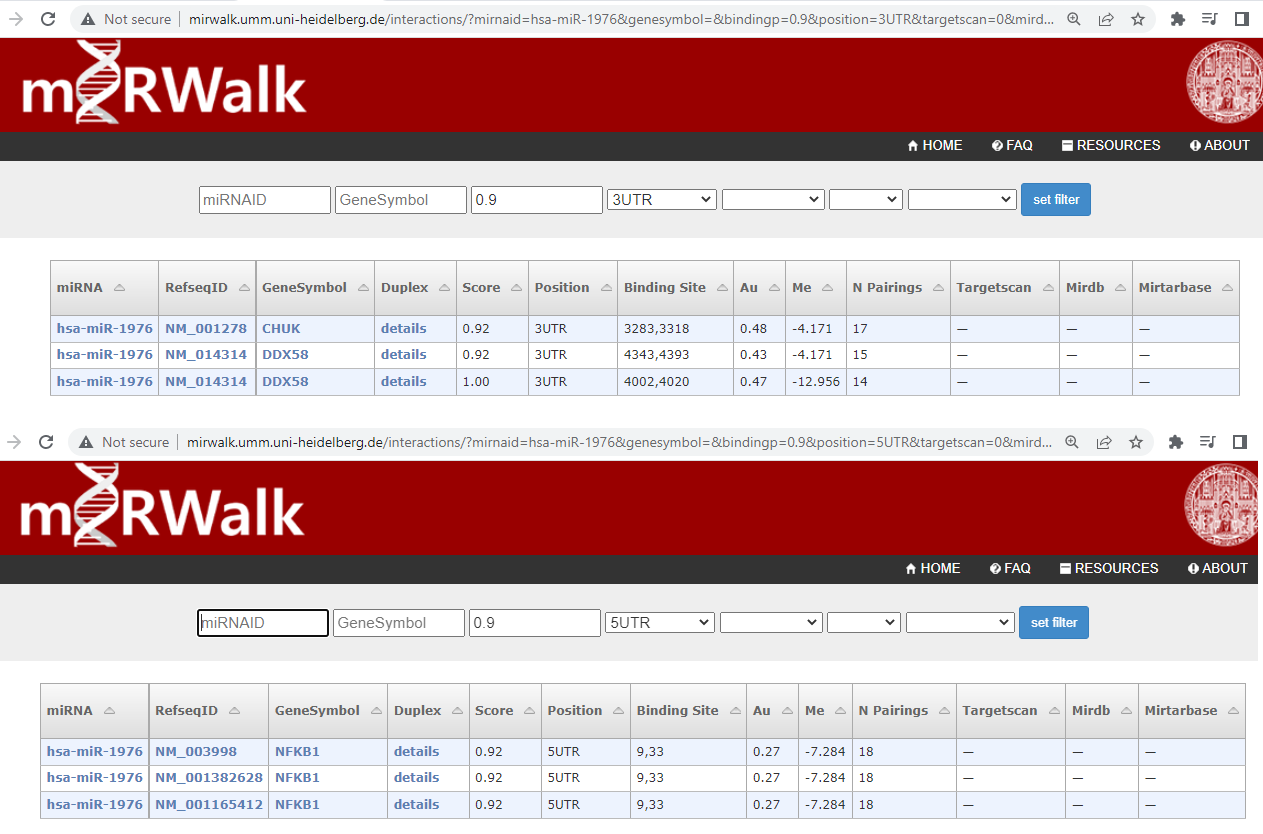
**

**S7 Figure:** Validation of the relation of miR-1976 to cGAS-STING-related pathways through DIANA tools mirPath 3 (<http://www.microrna.gr/miRPathv3>, accessed on 22 Oct 2021)

**
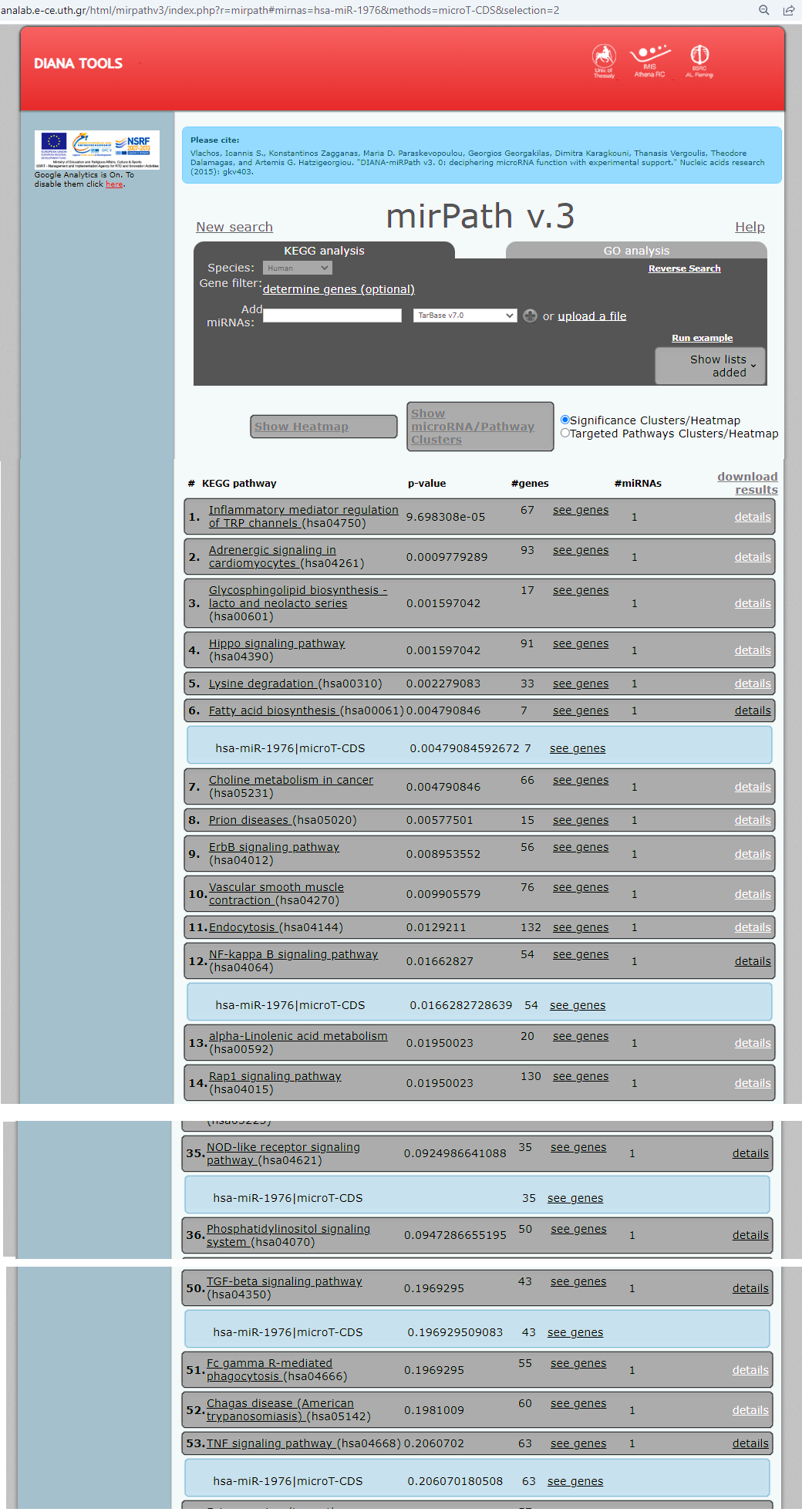
**

**Supplementary tables**

**S1 Table. :** List of primer assays.

| **Primer Assay** | **GeneGlobe ID** |
| --- | --- |
| DDX58 (NM_001106645) | QT00395731 |
| NFKB1 (NM_001276711) | QT00370545 |
| CHUK (NM_001107588) | QT01627724 |
| GAPDH (NM_017008) | PPR06557B-200 |
| miR-1976 | MI0009986 |
| SNORD72_11 | MS00033719 |
